# Triepoxide formation by a flavin-dependent monooxygenase in monensin biosynthesis

**DOI:** 10.1038/s41467-023-41889-0

**Published:** 2023-10-07

**Authors:** Qian Wang, Ning Liu, Yaming Deng, Yuze Guan, Hongli Xiao, Tara A. Nitka, Hui Yang, Anju Yadav, Lela Vukovic, Irimpan I. Mathews, Xi Chen, Chu-Young Kim

**Affiliations:** 1 Department of Chemistry and Biochemistry, The University of Texas at El Paso, 500 West University Avenue, El Paso, TX 79968 USA; 2 Key Laboratory of Synthetic and Natural Functional Molecular Chemistry of Ministry of Education, College of Chemistry and Materials Science, Northwest University, 710127 Xi’an, China; 3 Stanford Synchrotron Radiation Lightsource, SLAC National Accelerator Laboratory, 2575 Sand Hill Road, Menlo Park, CA 95124 USA; 4 Present Address: Department of Biochemistry, University of Illinois Urbana-Champaign, Urbana, IL 61801 USA

**Keywords:** Natural product synthesis, X-ray crystallography, Oxidoreductases, Enzyme mechanisms

## Abstract

Monensin A is a prototypical natural polyether polyketide antibiotic. It acts by binding a metal cation and facilitating its transport across the cell membrane. Biosynthesis of monensin A involves construction of a polyene polyketide backbone, subsequent epoxidation of the alkenes, and, lastly, formation of cyclic ethers via epoxide-opening cyclization. MonCI, a flavin-dependent monooxygenase, is thought to transform all three alkenes in the intermediate polyketide premonensin A into epoxides. Our crystallographic study has revealed that MonCI’s exquisite stereocontrol is due to the preorganization of the active site residues which allows only one specific face of the alkene to approach the reactive C(4a)-hydroperoxyflavin moiety. Furthermore, MonCI has an unusually large substrate-binding cavity that can accommodate premonensin A in an extended or folded conformation which allows any of the three alkenes to be placed next to C(4a)-hydroperoxyflavin. MonCI, with its ability to perform multiple epoxidations on the same substrate in a stereospecific manner, demonstrates the extraordinary versatility of the flavin-dependent monooxygenase family of enzymes.

## Abstract (web summary)

MonCI, a flavin-dependent monooxygenase, transforms all three C = C groups in the polyene substrate into epoxides during monensin A biosynthesis. Here, the authors present the structural basis for this enzyme’s regio- and stereoselective epoxidation activity.

## Introduction

Polycyclic polyether natural products have attracted much attention due to their wide-ranging biological activities that include antimicrobial, antifungal, and antitumor properties^1,2^. The majority of polycyclic polyethers discovered to date are of polyketide origin and are produced by actinomycete bacteria, while others are derived from terpenes and are produced by both terrestrial and marine organisms. Polyether natural products vary in the number, size, and arrangement of the cyclic ethers they contain. Ionophore polyethers (e.g., lasalocid, monensin, nanchangmycin) contain multiple five-to-six membered cyclic ethers connected by spiroketals or carbon-carbon bonds, whereas ladder polyethers (e.g., brevetoxin, ciguatoxin, maitotoxin) contain five-to-nine-membered cyclic ethers that are fused in a *trans*-*syn*-*trans* arrangement. In 1983, Cane, Celmer, and Westley proposed a unified model for ionophore polyether biogenesis which has been shown to be accurate for all natural polyethers studied to date^3^. According to this hypothesis, all ionophore polyethers are synthesized in the following manner: First, an *all-E* polyene is constructed from carboxylic acid building blocks by the cooperative action of type I modular polyketide synthases. Next, the polyene is oxygenated repeatedly by a monooxygenase to form the polyepoxide intermediate. Lastly, epoxides are regioselectively ring-opened by one or more epoxide hydrolases that involves an intramolecular nucleophilic attack on the epoxide groups^4^. In some instances, including monensin biosynthesis, additional tailoring reactions such as hydroxylation and methylation ensue to generate the final product. Recent studies suggest that the biosynthesis of ladder polyethers also follows this general pathway^5–7^.

Monensin A is a prototypical ionophore polyether antibiotic produced by the soil bacterium *Streptomyces cinnamonensis*^8,9^. It acts by chelating metal cations, such as Na^+^ and K^+^, rendering them membrane permeable and thereby disrupting the physiological ion concentration gradient across the cell membrane. Monensin A is widely used as an anticoccidial agent in poultry and cattle^10^. Biosynthesis of monensin has been studied extensively for half a century. In 1973, Day et al. used carbon isotope labeling to show that monensin A is synthesized from five acetate, seven propionate, and one butyrate precursors^11^. In 1982, Cane et al. used oxygen isotope labeling to demonstrate that three of the five ether oxygens (attached to C13, C17, and C21) in monensin are derived from molecular oxygen^12^. In 1983, Ajaz and Robinson showed that the oxygen atom attached to C26 is also derived from molecular oxygen^13^. In 2003, Oliynyk et al. reported the identification and analysis of the monensin biosynthetic gene cluster from *S. cinnamonensis*^14^. Monensin biosynthesis is accomplished by eight type I polyketide synthases, one monooxygenase, two epoxide hydrolases, one hydroxylase, and one methyltransferase. Oliynyk et al. also demonstrated that overexpression of *monCI* in a heterologous host increases its ability to epoxidize linalool, an unsaturated terpene, by as much as 20-fold, thereby establishing MonCI as the enzyme responsible for the epoxidation step in monensin biosynthesis^14^. In 2006, Gallimore et al. conducted gene deletion experiments which indicated that MonBI and MonBII have epoxide hydrolase activity^15^, which was later demonstrated directly by Minami et al. using purified recombinant proteins and a bisepoxide substrate analog^16^. Also in 2006, Harvey et al. determined that MonCII is a thioesterase responsible for releasing the final product from the acyl carrier protein (ACP)^17^. In 2014, Hüttel et al. confirmed the hydroxylation and methylation activity of MonD and MonE, respectively^18^.

**Fig. 1:**
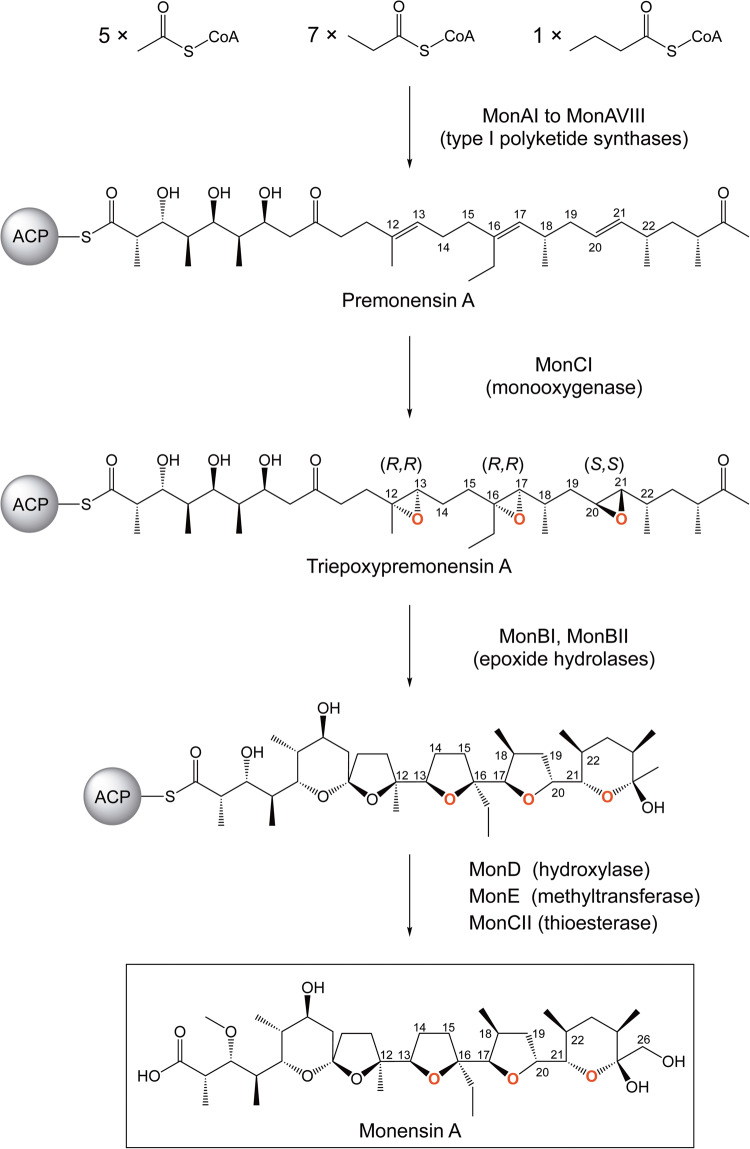
Monensin A biosynthesis in *Streptomyces cinnamonensis*. Oxygen atoms installed by MonCI are colored in red. During biosynthesis, polyketide intermediates remain attached to the acyl carrier protein (ACP) via a thioester bond.

The epoxidation step in monensin A biosynthesis is highly intriguing. A single monooxygenase enzyme, MonCI, is thought to act on premonensin A three times to produce the intermediate product triepoxypremonensin A (Fig. 1)^19^. Each epoxidation reaction is carried out in a highly stereospecific manner, thus exclusively producing the (12 *R*,13 *R*,16 *R*,17 *R*,20 *S*,21 *S*)-triepoxypremonensin A. We have conducted biochemical, structural, mutagenesis, and molecular dynamics simulation studies to elucidate the molecular basis of this unique enzymatic transformation. MonCI converted all three C = C groups in the model substrate farnesyl 4-hydroxybenzoate (FHB) into epoxides, providing experimental support for the hypothesis that it performs multiple epoxidations during monensin biosynthesis. Crystallographic analysis revealed that MonCI has an unusually large substrate binding pocket. This allows premonensin A to adopt at least three different poses in the active site cavity, thus enabling three separate epoxidations to take place on the same substrate molecule. Modeling study and molecular dynamics simulation showed that the prearrangement of MonCI’s substrate-binding pocket and the structure of the substrate molecule itself are responsible for the stereo- and regiocontrol exerted by the enzyme.

## Results

### Recombinant MonCI production and characterization

MonCI containing an N-terminal 6xHis-tag was expressed in *Escherichia coli* BL21-AI and purified using Ni-affinity, anion exchange, and size-exclusion chromatography. MonCI purified as a monomer with an apparent molecular weight of 53.8 kDa (theoretical molecular weight = 55.1 kDa) (Supplementary Fig. 1). The protein solution had an intense yellow color, indicating the presence of a flavin coenzyme, and its ultraviolet–visible spectrum showed a peak at 455 nm that is characteristic of oxidized flavin (Supplementary Fig. 2). Addition of sodium dithionite produced a colorless protein solution and the 455 nm peak disappeared, further confirming that MonCI is a flavoprotein and that the enzyme-bound flavin moiety can be readily reduced.

### MonCI activity assay

**Fig. 2:**
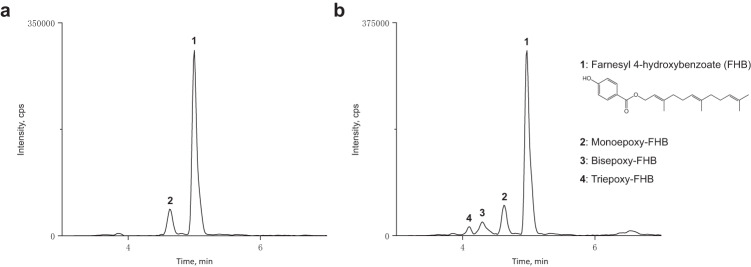
LC-MS analysis of the products formed by MonCI in the absence and presence of an NADH regeneration system. **a** Reaction mixture containing 160 µM farnesyl 4-hydroxybenzoate (FHB), 4 mM NADH, 15 µM Fre, 300 mM NaCl, 10% (v/v) glycerol, and 15 µM MonCI in 50 mM Tris, pH 8.0 was incubated for six hours at 30 °C prior to LC-MS analysis. **b** Reaction mixture containing 160 µM farnesyl 4-hydroxybenzoate, 15 µM glucose dehydrogenase, 80 mM d-glucose, 4 mM NAD^+^, 15 µM Fre, 300 mM NaCl, 10% (v/v) glycerol, and 15 µM MonCI in 50 mM Tris, pH 8.0 was incubated for 6 h at 30 °C prior to LC-MS analysis. Source data are provided as a Source Data file. Mass spectrum corresponding to individual peaks are presented in Fig. 3.

The native substrate of MonCI is a tridecaketide tethered to an ACP (Fig. 1). Since this substrate is not readily accessible, we synthesized and used a substrate analog, FHB, to probe the catalytic activity of recombinant MonCI (Supplementary Fig. 3). The reaction mixture contained 160 µM FHB, 4 mM NADH, 15 µM flavin reductase (Fre), 300 mM NaCl, 10% (v/v) glycerol, and 15 µM MonCI in 50 mM Tris, pH 8.0. Exogenous FAD was added to compensate for the potentially weak binding of FAD to MonCI. Addition of FAD was shown to increase product formation for other monooxygenases^20,21^. Fre was included because it was reported to enhance the activity of Lsd18, the flavin-dependent monooxygenase involved in lasalocid A biosynthesis^22^. Fre is thought to act on free FAD and thus promote saturation of the monooxygenase active site with reduced FAD. The reaction mixture was incubated for 6 h at 30 °C and then subjected to LC-MS analysis. A major peak corresponding to the theoretical mass of the unreacted FHB and a minor peak corresponding to the theoretical mass of a monoepoxide product was observed (Fig. 2a). Reversed-phase high-performance liquid chromatography (HPLC) analysis showed that MonCI has similar preference for NADH and NADPH, and addition of Fre increased the product yield by about 20% (Supplementary Fig. 4). As expected, no product was detected when the reaction mixture excluded MonCI or NAD(P)H (Supplementary Fig. 5).

To further confirm the identity of the MonCI reaction product, we compared its chemical and physical property to that of the chemically synthesized 17,18-epoxy-FHB standard (Supplementary Fig. 6). First, we performed a colorimetric assay using 4-(4-nitrobenzyl)pyridine (NBP)^23^. The product solution became purple colored upon addition of NBP, indicating the presence of an epoxide (Supplementary Fig. 7). Next, we conducted HPLC analysis of the 17,18-Epoxy-FHB standard and the MonCI reaction product (Supplementary Fig. 8). The retention times were nearly identical, which suggested that MonCI had indeed produced the monoepoxide product. However, we were unable to generate enough of the product compound to conduct a detailed NMR spectroscopic analysis.

In general, flavin-dependent monooxygenases capture molecular oxygen to form a reactive C(4a)-hydroperoxyflavin species during the oxidative half-reaction, which in turn incorporates one oxygen atom into the substrate molecule while the other oxygen atom is reduced to water^24–26^. Recently, it was reported that the monooxygenase involved in enterocin and tropolone biosynthesis utilize flavin-N5-oxide rather than the canonical C(4a)-hydroperoxyflavin^27–29^. These monooxygenases are also unique in that they use their own substrate as the electron donor instead of NAD(P)H. We measured the ultraviolet–visible spectrum of MonCI immediately following purification from the *E. coli* lysate and after a 12-h incubation with substrate and cofactors (Supplementary Fig. 9). In both cases, we did not observe an absorbance peak at 460 nm that would indicate the presence of flavin-N5-oxide^27^. Therefore, we predict that MonCI utilizes C(4a)-hydroperoxyflavin for substrate epoxidation (Supplementary Fig. 10). However, further studies are needed to conclusively exclude the involvement of flavin-N5-oxide.

### Epoxidation with NADH regeneration

**Fig. 3:**
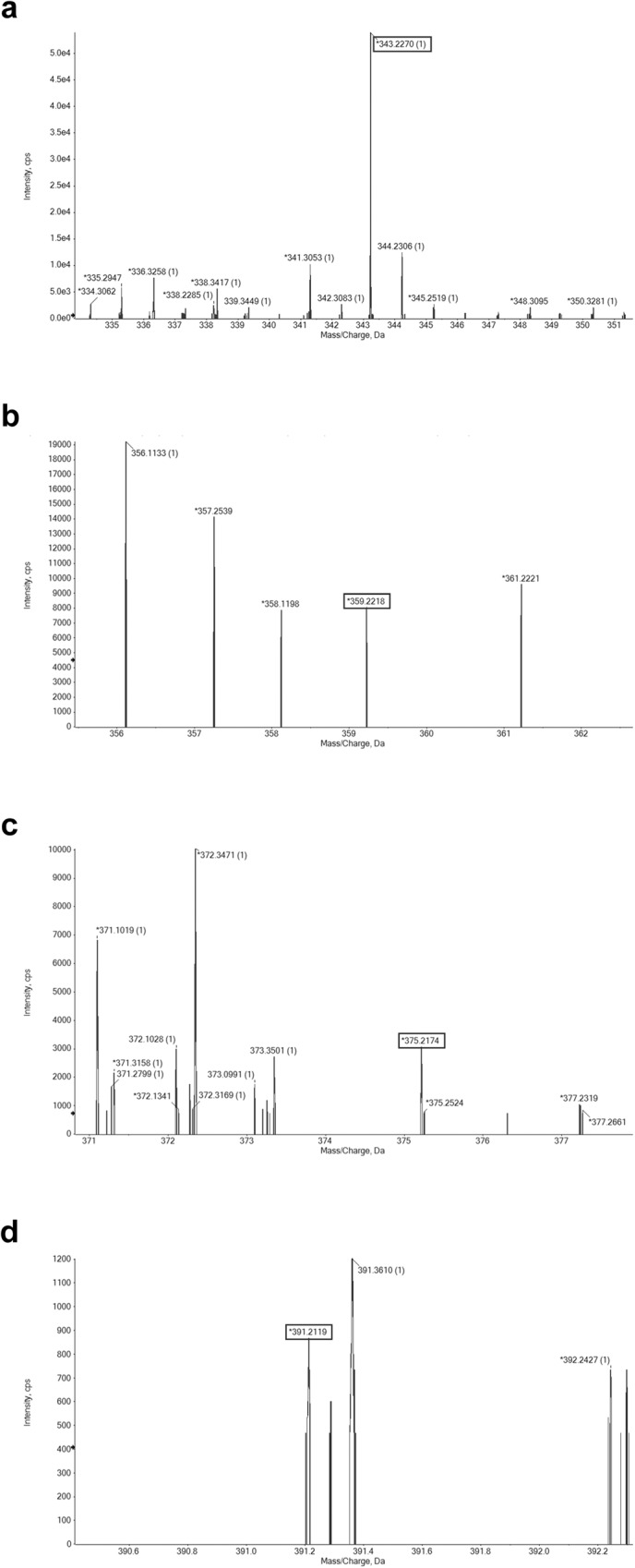
Positive ion ESI-MS spectra of MonCI reaction products. MonCI produces single, double, and triple epoxidation products of farnesyl 4-hydroxybenzoate. Mass spectra of **a** Peak 1 (Fig. 2b) contained mass corresponding to unreacted farnesyl 4-hydroxybenzoate (theoretical [M-H]^+^ = 343.2267 *m/z*). **b** Peak 2 (Fig. 2b) contained mass corresponding to a monoepoxide product (theoretical [M-H]^+^ = 359.2216 *m/z*). **c** Peak 3 (Fig. 2b) contained mass corresponding to a bisepoxide product (theoretical [M-H]^+^ = 375.2166 *m/z*). **d** Peak 4 (Fig. 2b) contained mass corresponding to the triepoxide product (theoretical [M-H]^+^ = 391.2116).

We were puzzled by the observation that MonCI only generated the monoepoxide product and not the bisepoxide and triepoxide products. To test if NADH was the limiting factor, we implemented an NADH regeneration system in our in vitro reaction assay^30,31^. Reaction mixture containing 160 µM FHB, 15 µM glucose dehydrogenase, 80 mM D-glucose, 4 mM NAD^+^, 15 µM Fre, 300 mM NaCl, 10% glycerol, and 15 µM MonCI in 50 mM Tris, pH 8.0 was incubated for 6 h at 30 °C and then subjected to LC-MS analysis. This time, four peaks were observed. The largest peak contained mass corresponding to the unreacted FHB, the second largest peak contained mass corresponding to the monoepoxide product, the third largest peak contained mass corresponding to the bisepoxide product, and the smallest peak contained mass corresponding to the triepoxide product (Fig. 2b, Fig. 3 and Supplementary Fig. 11). This result showed that MonCI can perform multiple epoxidations on the same substrate molecule, as it had been predicted based on the observation that *monCI* is the only monooxygenase gene found in the monensin biosynthetic gene cluster. However, the order of the epoxidation reactions and whether the substrate remains bound to MonCI in between epoxidation reactions remain unknown.

### X-ray crystal structure of MonCI

To elucidate the structural basis of MonCI’s catalytic activity, we determined the MonCI crystal structure at 1.9 Å resolution using the multi-wavelength anomalous diffraction method (Supplementary Table 1). Two molecules of MonCI are present in the crystal asymmetric unit (chain A and B). Some sections of chain A (residues 1–3 and 481–496) and chain B (residues 1–3, 147–149, 230–232, and 478–496) could not be modelled due to lack of electron density. One FAD coenzyme and one chloride ion were found in each chain (Supplementary Fig. 12). The final MonCI model has 7,180 protein atoms, 106 ligand atoms, 2 chloride ions, 721 water molecules, and *R*_work_ and *R*_free_ of 0.18 and 0.22, respectively.

**Fig. 4:**
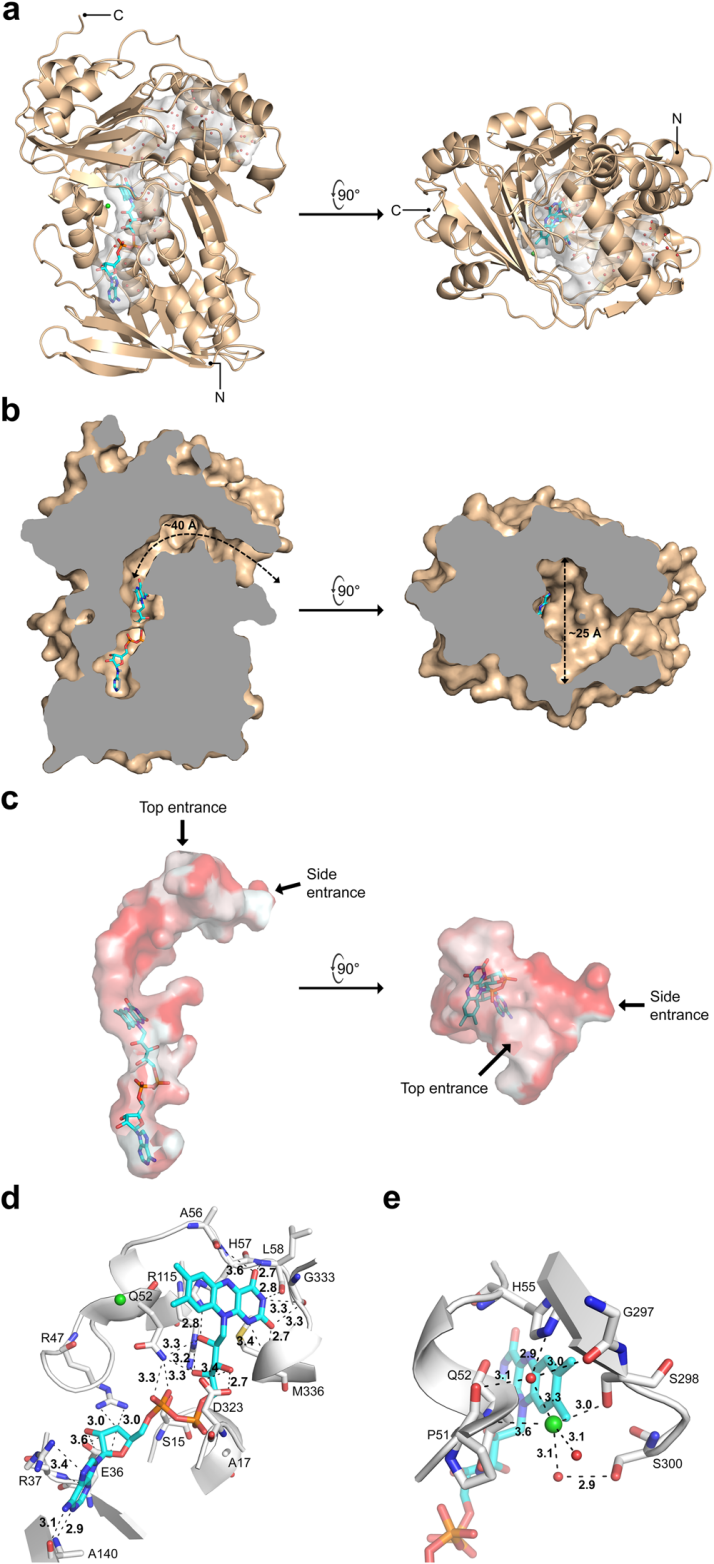
X-ray crystal structure of MonCI. Chloride ion and water are shown as green and red spheres, respectively. **a** Overall structure of MonCI. The FAD cofactor is drawn as a stick model. The fused FAD and substrate binding pocket is shown as a grey surface. For clarity, only the water molecules located inside this pocket are shown. **b** Cross-section of MonCI showing the dimensions of the substrate binding pocket. The indicated pocket length (~40 Å) is that of the pocket trajectory leading to the side entrance. **c** Top and side active site entrances that provide access to MonCI’s substrate binding pocket. Hydrophobic surfaces are colored in red and hydrophilic surfaces are colored in white. **d** Specific interactions involving the FAD cofactor. **e** Specific interactions involving the chloride ion.

MonCI consists of a β-strand-rich substrate-binding domain located near the C-terminus and a Rossmann-like three-layer ββα sandwich FAD-binding domain located near the N-terminus (Fig. 4a). These two domains are extensively integrated, with multiple noncontiguous segments of the protein sequence contributing to each domain. The FAD- and substrate-binding pockets are fused and form a continuous chamber that is shaped like an inverted letter L (Fig. 4b). The substrate-binding pocket, which lies immediately on top of the isoalloxazine ring of FAD, is about 40 Å long and about 25 Å wide. Structure alignment using the DALI server^32^ identified the following proteins as the closest MonCI structural homologs: bacterial halogenase Bmp2 (RMSD = 2.9 Å)^33^, human squalene epoxidase (RMSD = 3.0 Å)^34^, and bacterial *p*-hydroxybenzoate hydroxylase (RMSD = 3.4 Å)^35^ (Supplementary Fig. 13). These monooxygenases share 13–18% sequence identity with MonCI. In general, the size of the substrate-binding pocket in monooxygenases correlates with the size of their native substrate. Interestingly, MonCI has an unexpectedly large substrate binding pocket. The volume of the fused FAD- and substrate-binding pocket of MonCI is 995 Å^3^, as calculated using the program CASTp 3.0^36^. By comparison, the pocket volume of squalene epoxidase is only 629 Å^3^, even though the size of squalene and premonensin A are comparable. The significance of MonCI’s oversized substrate binding pocket is discussed in a later section.

MonCI has two different entrances through which the substrate and solvent molecules can potentially enter and exit the enzyme, namely a side entrance and a top entrance (Fig. 4c). These entrances are orthogonally positioned, and they both have an opening with a diameter of about 6.5 Å. While the side entrance is connected to the active site cavity by a narrow, 8.5 Å-long tunnel, the top entrance leads directly to the active site cavity. We predict that premonensin A enters MonCI via the side entrance based on the following three observations. First, MonCI is known to act on premonensin A which is tethered to a carrier protein, MonACPX^37^. Hence, MonACPX must dock to MonCI before catalysis can occur. ACPs are known to interact with their partner proteins primarily by electrostatic interaction^38–41^. We generated a homology model of the MonACPX using the program MODELLER^42^. In this model, Ser60, which is the attachment site of the 4´-phosphopantetheine prosthetic group, is located next to Glu55 and Glu65, resulting in an electrostatically negative protein surface. Our MonCI crystal structure shows that the surface surrounding the side entrance is electrostatically positive whereas the surface surrounding the top entrance is largely negative (Supplementary Fig. 14). Therefore, the side entrance of MonCI is more favorable for MonACPX docking than the top entrance. Second, the location of the substrate entrance in other flavin-containing monooxygenases aligns better with MonCI’s side entrance than its top entrance. For example, the cavity leading to the side entrance in MonCI aligns well with the way squalene is thought to bind to the human squalene monooxygenase^34^, a close structural homolog of MonCI (Supplementary Fig. 15). These observations suggest that premonensin A enters MonCI via the side entrance. We speculate that the top entrance in MonCI functions as a solvent channel. A similar two-entrance system was previously reported for 3-hydroxybenzoate hydroxylase from *Comamonas testosteroni*^43^. Third, the protein surface near the side entrance is lined with hydrophobic residues, presumably enhancing the interaction with the greasy premonensin A, while the surface near the top entrance is lined with hydrophilic residues, promoting interaction with water (Fig. 4c).

FAD is bound noncovalently to MonCI through numerous direct and solvent-mediated interactions (Fig. 4d). The isoalloxazine ring of FAD makes a π-π stacking interaction with the imidazole group of His57, and the N1, N3, O2, and O4 atoms form hydrogen bonds with the backbone atoms of Ala56, Leu58, Gly333, and Met336, respectively. The pyrophosphate group of FAD forms hydrogen bonds with the Ser15 side chain and Ala17 main chain atoms, and the adenine base of FAD forms a π-cation interaction with the guanidine group of Arg37 and hydrogen bond interaction with Ala140. One chloride ion was modelled next to the isoalloxazine ring of FAD. The chloride ion coordinates with five hydrogen bond donors (Gln52 main chain nitrogen, Ser298 side chain hydroxyl group, and three water molecules) in a distorted trigonal bipyramidal stereochemistry (Fig. 4e). Structural alignment of the MonCI (PDB ID: 8T3P) and the 4-hydroxybenzoate hydroxylase (PDB ID: 1K0J) crystal structures shows that the chloride in MonCI is located where the adenine base of NAD(P)H is expected to bind (Supplementary Fig. 16)^44^. Therefore, chloride is expected to act as a competitive inhibitor of MonCI. In agreement with this theory, chloride was previously shown to inhibit the flavoenzyme *p*-hydroxybenzoate hydroxylase, a structural homolog of MonCI, although the inhibition mechanism was not determined^45^.

Flavin-dependent monooxygenases can be categorized into eight subclasses (A to H)^46,47^. MonCI is a class A flavin-dependent monooxygenase based on its structural and biochemical features. Like other monooxygenases in this class, MonCI contains a bound FAD, requires NAD(P)H for activity, and lacks a discrete dinucleotide-binding domain, indicating that MonCI binds NAD(P)H transiently. Class A monooxygenases are thought to follow a ping-pong type mechanism, where NAD(P)H only binds in the presence of substrate and NAD(P)^+^ is released prior to addition of oxygen to the substrate^48^. This is made possible by an ~8 Å swinging movement of the flavin isoalloxazine ring^21,49^. In the “in” conformation, the isoalloxazine ring is located immediately next to the substrate, while in the “out” conformation, the isoalloxazine ring is positioned away from the substrate-binding pocket for interaction with NAD(P)H. Based on the structural similarity of MonCI with other class A monooxygenases that have been experimentally shown to employ flavin movement, especially *p*-hydroxybenzoate hydroxylase, we predict that the FAD in MonCI also undergoes such movement during catalysis (Supplementary Fig. 17). In our MonCI crystal structure, the FAD is in the “in” conformation, which is the expected conformation during epoxidation.

### Interaction between MonCI and MonACPX

**Fig. 5:**
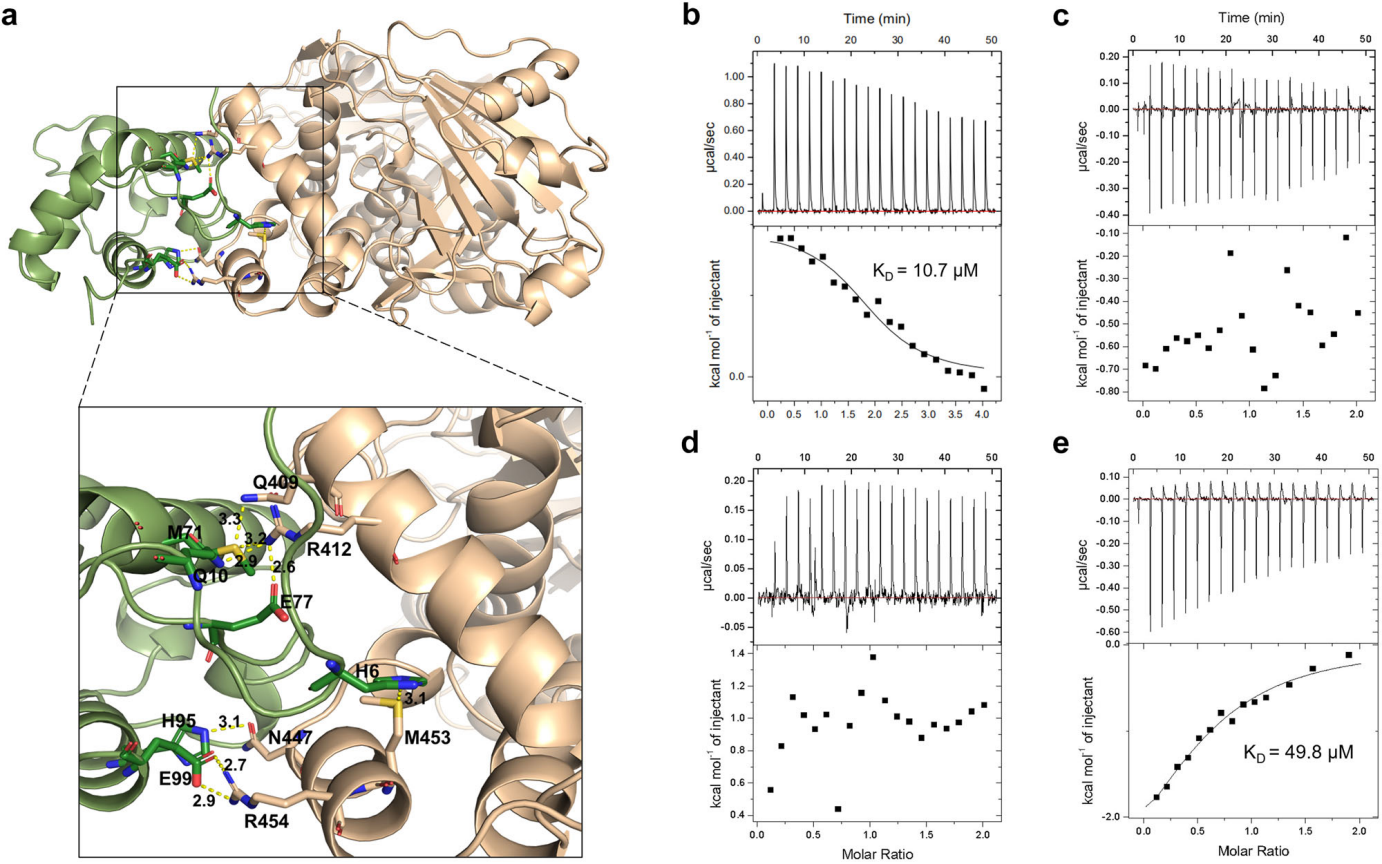
MonCI–MonACPX interaction. **a** Predicted structure of the MonCI-MonACPX complex. MonCI is shown in gold and MonACPX is shown in green. **b** Isothermal titration calorimetry profile of wild-type MonACPX binding to MonCI. **c** E77A MonACPX binding to MonCI. **d** E99A MonACPX binding to MonCI. **e** DEBS ACP2 binding to MonCI. Source data are provided as a Source Data file.

MonCI’s native substrate is a tridecaketide attached to the 4´-phosphopantetheine group of MonACPX via a thioester linkage. Therefore, formation of the MonCI-MonACPX complex is a prerequisite for the MonCI-catalyzed epoxidation reaction. To study how this complex is formed, we docked the MonACPX homology model to the MonCI crystal structure using the program HADDOCK^50^ (Fig. 5a). This structure showed presence of two salt bridges, formed by Arg412 and Arg454 of MonCI with Glu77 and Glu99 of MonACPX, respectively. Glu77 resides on a loop while Glu99 resides on helix 6 of MonACPX. Isothermal titration calorimetry showed that the interaction between MonCI and wild-type *apo*-MonACPX is entropy-driven and is slightly endothermic (Fig. 5b). This interaction had a dissociation constant of 10.7 µM, which is in line with previously reported values for other *apo*-ACPs^51,52^. Mutating either Glu77 or Glu99 of MonACPX to Ala abolished binding (Fig. 5c, d). Next, we tested if MonCI could bind to a non-native ACP partner. Here, we used *apo*-DEBS ACP2 from module 2 of the 6-deoxyerythronolide B, a type I polyketide synthase^53^. The interaction between MonCI and DEBS ACP2 was exothermic and had a dissociation constant of 49.8 µM (Fig. 5e). DEBS ACP2 has an Arg at the position equivalent to Glu77 of MonCI, and a Glu at the position equivalent to Glu99 of MonCI (Supplementary Fig. 18). Overall, MonCI bound to its native ACP partner five times more strongly than to DEBS ACP2, indicating that ACP interaction plays an important role in determining the substrate specificity of MonCI.

### Molecular basis for the stereoselectivity of MonCI

**Fig. 6:**
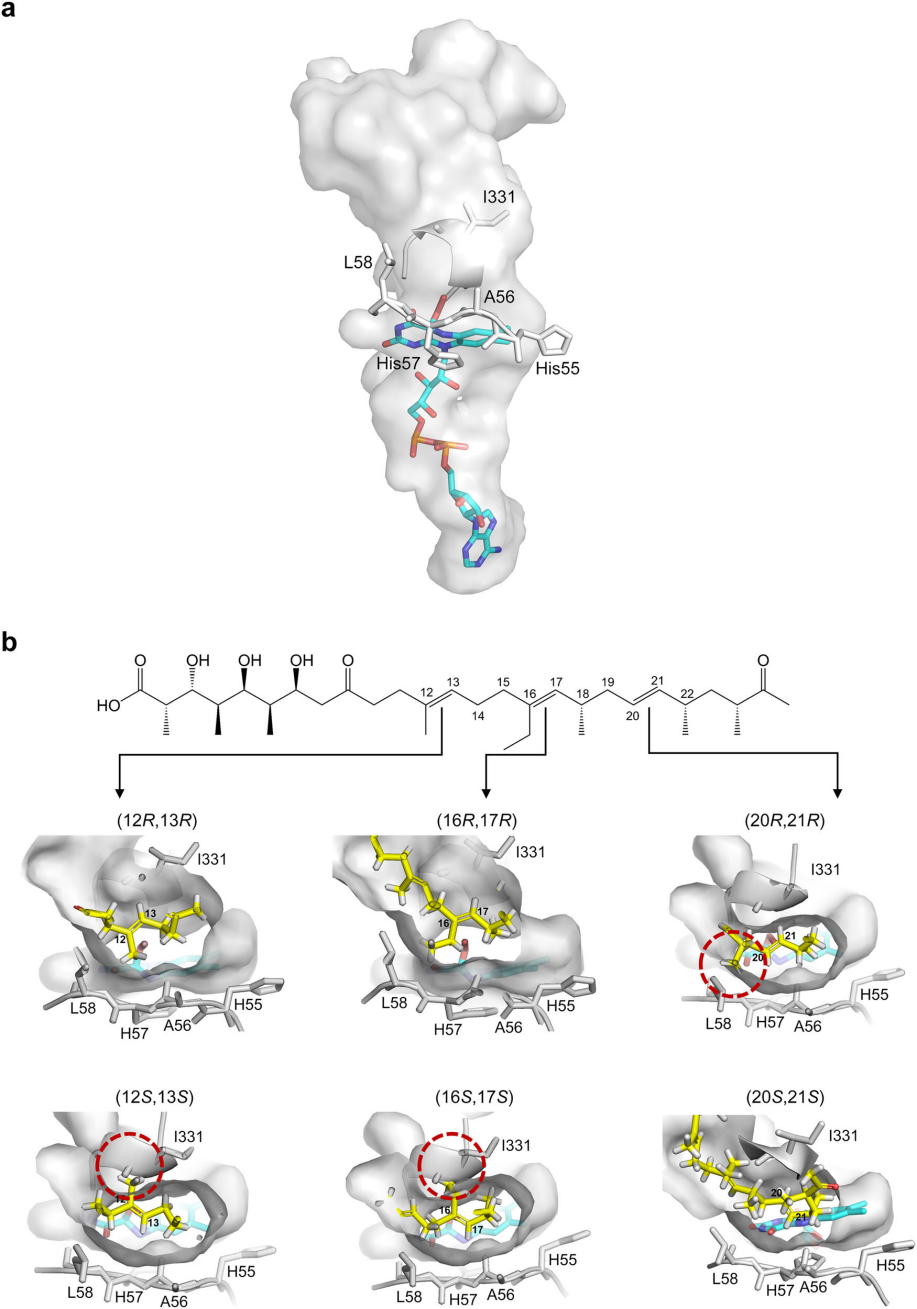
Stereoselectivity of MonCI. **a** The fused substrate- and FAD-binding pocket of MonCI and the predicted structure of the bound C(4a)-(hydro)peroxyflavin adenine dinucleotide. **b** Six models of the MonCI–premonensin A complex. Premonensin A was manually built inside the MonCI pocket in the conformation required to produce each of the six stereoisomers. Red circles indicate regions of steric clash between MonCI and premonensin A.

Epoxidation of all three C = C groups in premonensin A can theoretically produce eight different triepoxypremonensin A stereoisomers (Supplementary Fig. 19). Remarkably, MonCI exclusively produces the (12 *R*,13 *R*,16 *R*,17 *R*,20 *S*,21 *S*)-triepoxypremonensin A product. To elucidate the molecular basis for this exquisite stereoselectivity, we conducted a modeling study based on the MonCI crystal structure. First, we modified the FAD molecule in the crystal structure to the reactive C(4a)-hydroperoxyflavin. Here, we had the option of placing the peroxide group either on the *re* or *si* face of C4a of the isoalloxazine ring. In our MonCI crystal structure, the *si* face is packed against a loop (residues 55–58) and so it is unlikely for molecular oxygen to approach the isoalloxazine ring from this side. In contrast, the *re* face is fully exposed to the water-filled cavity, and therefore oxygen is likely to approach the flavin’s C4a atom from the *re* side. Therefore, we generated the structure of MonCI containing the C(4a)-hydroperoxyflavin intermediate in which the peroxide group protrudes out from the *re* face (Fig. 6a).

Next, we manually constructed atomic models of premonensin A inside the MonCI active site pocket using the program Coot^54^. We assumed that MonCI’s pocket is rigid. Since we did not know the order in which the three epoxidations take place inside the cell, we decided to model only the first epoxidation event where the substrate is unequivocally the fully unepoxidated premonensin A. This initial epoxidation reaction could take place at any one of the three positions (C12 = C13, C16 = C17, C20 = C21), and the oxygen atom could be added to either face of the alkene. Therefore, we modeled enzyme-substrate complexes that reflect each of the six possible scenarios. The only restraint we applied was that the C = C group that undergoes epoxidation must lie within 6 Å of the hydroperoxy group of C(4a)-hydroperoxyflavin. We found that steric clash with active site residues prevents the alkene to be placed adjacent to the hydroperoxy group in three of the six possible cases (Fig. 6b). The substrate could be placed inside the active site pocket in a conformation that is conducive to forming the (12 *R*,13 *R*)-, (16 *R*,17 *R*)-, and (20 *S*,21 *S*)-epoxide. However, positioning the substrate in a conformation that is conducive to forming the (12 *S*,13 *S*)-, (16 *S*,17 *S*)-, and (20 *R*,21 *R*)-epoxide resulted in a steric clash. For (12 *S*,13 *S*)-epoxide formation, there is a potential clash between 12-Me of premonensin A and the main chain of Ile331. For (16 *S*,17 *S*)-epoxide formation, there is a potential clash between 16-Et of premonensin A and the main chain of Ile331. For (20 *R*,21 *R*)-epoxide formation, there is a potential clash between 18-Me of premonensin A and the side chain of Leu58. Incidentally, the three favorable stereochemical outcomes we have identified through the modeling exercise match the stereochemistry of the natural intermediate (12 *R*,13 *R*,16 *R*,17 *R*,20 *S*,21 *S*)-triepoxypremonensin A. Based on these observations, we propose that the active site pocket shape dictates the binding conformation of premonensin A, which in turn determines the stereochemical outcome of the epoxidation reaction. It is important to note that the structure of the substrate molecule also plays an important role in this type of stereocontrol mechanism. The steric clashes we have identified in the modeling study involve the 12-Me, 16-Et and 18-Me groups of premonensin A (Fig. 6b). Therefore, any modification of the substrate at these positions could alter the stereochemical outcome of the MonCI catalyzed epoxidation reaction. For example, deletion of the said methyl or ethyl group will likely result in production of a mixture of epoxide stereoisomers.

All ionophore polyether biosynthetic gene clusters discovered to date contain a gene that codes for a MonCI homolog. For example, nanchangmycin gene cluster contains *nanO*^55^, salinomycin gene cluster contains *salC*^56^, tetronomycin gene cluster contains *tmnC*^57^, and the lasalocid A gene cluster contains *lsd18*^58^. All these genes encode a flavin-dependent monooxygenase that, like MonCI, is expected to perform multiple epoxidations on a linear polyene polyketide substrate, setting them apart from other flavoenzymes. These polyether-producing epoxidases share a high sequence identity (49 to 60%). Sequence alignment analysis of MonCI, NanO, SalC, TmnC, and Lsd18 has revealed two highly conserved regions; RKGX^1^PQX^2^RHX^3^HX^4^LWSX^5^GA (residues 47–64 in MonCI; X^1^ = V/Q, X^2^ = A/G, X^3^ = A/V, X^4^ = L/V/I, X^5^ = N/S/G) and AFNPX^1^X^2^GHGMSX^3^X^4^A (residues 327–340 in MonCI; X^1^ = I/V, X^2^ = Y/H, X^3^ = S/C/V/A, X^4^ = A/S) (Supplementary Table 2). The former sequence is part of an extended loop located on the *si* side of C4a of the isoalloxazine ring, while the latter sequence is part of an α-helix located on the *re* side (Supplementary Fig. 20). Interestingly, Leu58 and Ile331, the two residues that we have identified in MonCI to play an important role in determining the stereochemical outcome are in these conserved regions. Therefore, other polyether-producing monooxygenases may utilize a similar steric-driven stereocontrol mechanism. It should be noted that the two conserved sequences we have identified are present only in flavin-dependent monooxygenases involved in polyether polyketide natural product biosynthesis.

### Molecular basis for the regioselectivity of MonCI

**Fig. 7:**
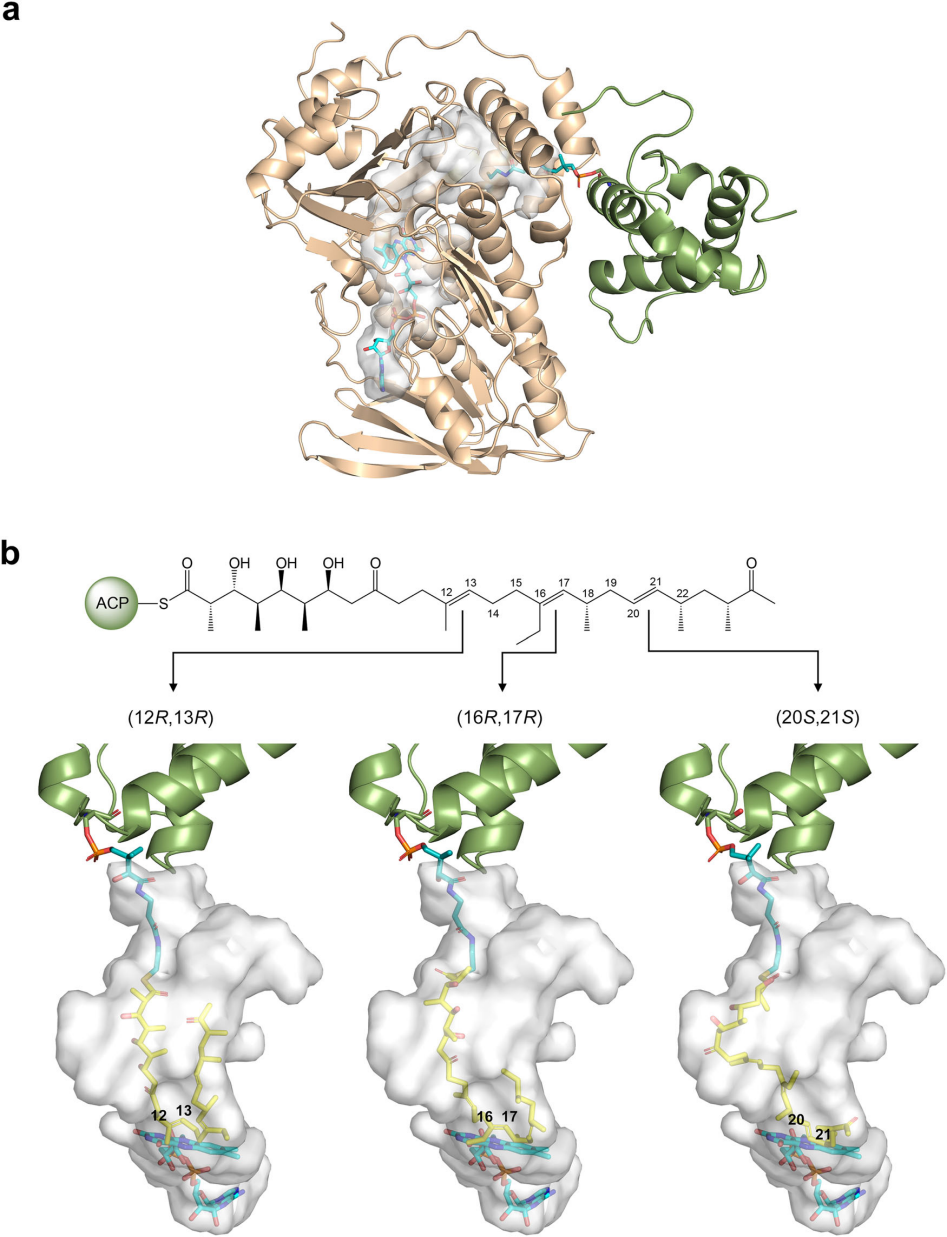
Regioselectivity of MonCI. **a** Predicted structure of the *holo*-MonACPX-MonCI complex. MonACPX and MonCI are colored in green and gold, respectively. **b** Predicted structure of the MonCI-premonensin A-MonACPX complex. Premonensin A was modelled inside the MonCI substrate-binding pocket by positioning each of the three double bonds next to the C4a atom of the isoalloxazine ring. The carbon atoms of 4´-phosphopantetheine, premonensin A, and FAD are colored in green, yellow, and light blue, respectively.

MonCI is thought to convert all three C = C groups in premonensin A into epoxides^19^. Since MonCI contains only one FAD, it can only catalyze one epoxidation at a time. For MonCI to transform all three C = C groups, the FAD must be reduced and then react with molecular oxygen in between epoxidation reactions. In addition, either MonCI or the substrate or both must undergo structural reorganization so that an alternate C = C is placed next to C(4a)-hydroperoxyflavin to enable the ensuing epoxidation reaction. We conducted a modeling study to understand how premonensin A may reorganize itself inside the binding pocket of MonCI while it is tethered to MonACPX. First, we attached the 4´-phosphopantetheine prosthetic group to the conserved Ser60 of MonACPX in our MonCI-MonACPX complex model (Fig. 7a). Because polyketide intermediates are tethered to the 4´-phosphopantetheine group of ACPs via a thioester linkage, our *holo*-MonACPX-MonCI model provides a good approximation of the location of the C1 atom of premonensin A in the actual enzyme-substrate complex. Then we placed premonensin A inside the MonCI active site cavity in three different conformations, each representing epoxidation of a different double bond in premonensin A and in the orientation that would yield the correct stereoisomer (Fig. 7b). Here, we imposed three constraints. (1) MonCI and MonACPX are static, (2) ACP and the phosphate group of the 4´-phosphopantetheine prosthetic group are immobile, and (3) the C = C group in premonensin A that undergoes epoxidation must lie within 6 Å of the C(4a)-hydroperoxyflavin. Our results showed that the substrate binding pocket of MonCI, due to its unusually large size, can accommodate premonensin A in three different conformations necessary for epoxidation of C12 = C13, C16 = C17, and C20 = C21. However, it is currently unknown if premonensin A leaves the enzyme after each epoxidation and then re-enters the enzyme for subsequent epoxidations, or if it leaves the enzyme only after all three epoxidations have been performed. Further studies are needed to determine the nature of the transitions which take place in between epoxidations.

### Molecular dynamics study of the MonCI–premonesin A complex

We performed atomistic molecular dynamics simulations to investigate the dynamics of the ligand-free MonCI, premonensin A (free acid) bound MonCI, and C12-C13 epoxidized substrate (free acid) bound MonCI. Premonensin A, when placed inside MonCI, is expected to take on a short and wide form as it folds into a U-shape, a longer and narrower J-shape, or a P-shape, depending on which C = C group is positioned at the active site. To examine if premonensin A in different shapes and orientations can fit stably within MonCI’s active site cavity, RMSDs of substrate-binding channel residues and RMSDs of substrate-FAD complexes were evaluated (Supplementary Fig. 21). In all the systems in which premonensin A was present, RMSDs of channel residues are smaller than 1.8 Å, and are slightly smaller than the RMSD for the ligand-free MonCI system. Therefore, the MonCI cavity design is such that it remains stable and robust while accommodating the substrate in all different shapes and orientations examined. Interestingly, average RMSDs of channel residues for native systems, with substrate positioned to generate the (12 *R*,13 *R*), (16 *R*,17 *R*) and (20 *S*,21 *S*) epoxides were lower than RMSDs of non-native poses that would generate the opposite configurations, although the differences are within standard deviations.

**Fig. 8:**
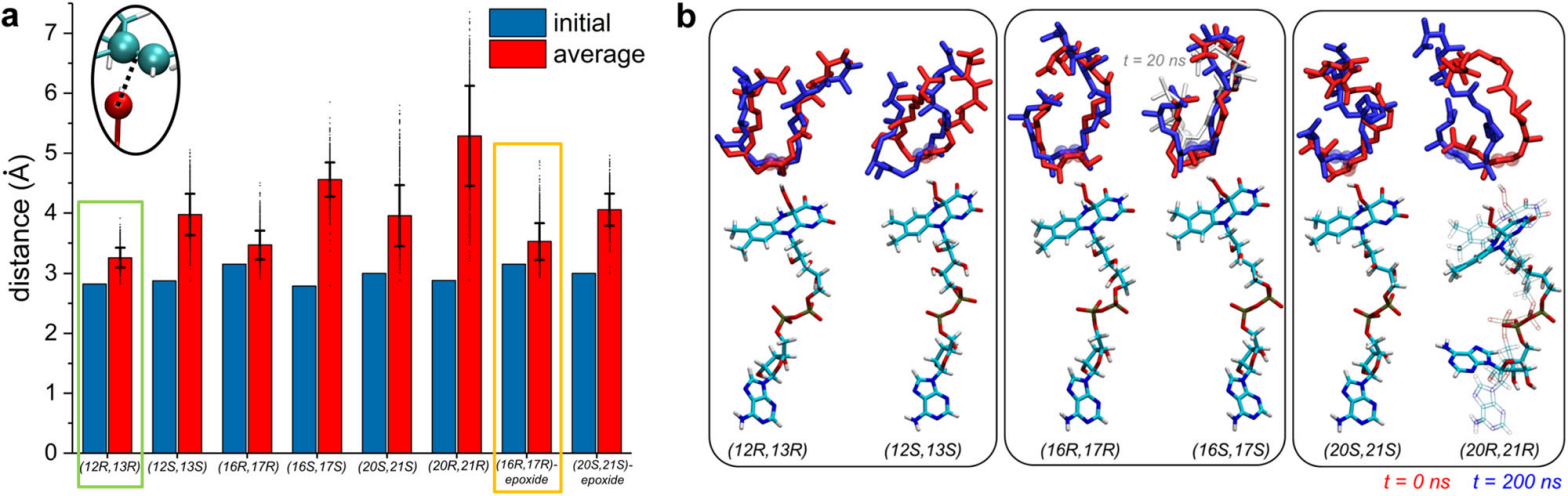
Molecular dynamics simulations of MonCI–premonensin A free acid. **a** Initial and average distances between the C = C bond and the distal oxygen atom of C(4a)-hydroperoxy group for each simulated system. Data are presented as mean values ± SD (*n* = 1000 structures extracted from the last 100 ns of one MD trajectory). **b** Conformation of premonensin A free acid at the start (red) and after 200 ns (blue) of simulation. Carbon atoms of the double bond nearest to the flavin group are shown as transparent spheres. In the (12 *R*,13 *R*) system, these carbon atoms are not displaced after 200 ns. In other systems, these carbon atoms become more displaced from their initial positions. Source data are provided as a Source Data file.

Epoxidation reactions of premonensin A in MonCI could occur in a predetermined order. In some cases, probability of a reaction taking place has been shown to depend on the stability of the substrate within the enzyme’s active site pocket^59–62^. The stability of premonensin A in different initial conformations within the MonCI active site cavity was tracked during 200 ns of molecular dynamics simulations, by evaluating distances between the C = C group and the distal oxygen atom of the C(4a)-hydroperoxy group (Fig. 8). In the case of the systems where C12 = C13 is initially near FAD, the substrate in the *R,R* configuration, but not the *S,S* configuration, remains very close to its initial conformation and the binding pose. Furthermore, the C12 = C13 group remains in a pose in which a chemical reaction is favored, within 3.3 Å of the distal oxygen atom of C(4a)-hydroperoxy moiety. In the systems where C16 = C17 and C20 = C21 are initially near FAD, substrates change shapes, and the C = C bonds move away from the flavin group. Overall, C12 = C13 moves away from the reaction site less than C16 = C17 and C20 = C21, and hence C12 = C13 is the more likely double bond to be epoxidized first. Tracking the stability of C12-C13 epoxidized substrate in MonCI active site further suggests that epoxidation proceeds at C16 = C17 in (16 *R*,17 *R*) configuration and then C20 = C21 in (20 *S*,21 *S*) configuration, since C16 = C17 and reactive flavin moiety stay closer to each other within the active site ( ~ 3.5 Å) than C20 = C21 and the reactive flavin moiety (~4.0 Å).

## Discussion

The class A monooxygenases characterized to date utilize a similar mechanism for installing an oxygen atom in their respective substrate molecule. The stereochemical outcome and the position of the carbon atom(s) of the substrate that is oxygenated depends on the orientation of the substrate with respect to the reactive flavin in the enzyme’s active site. Generally, the substrate orientation is dictated by specific interactions formed between the substrate molecule and the protein residues lining the active site pocket. However, MonCI employs a different strategy in which careful coordination of the hydrophobic packing interaction between the aliphatic premonensin A and binding cavity residues controls the substrate orientation. Furthermore, MonCI’s oversized substrate-binding pocket permits premonensin A to move and reorient while it is bound to the enzyme, thereby facilitating three epoxidations at three different positions of the same substrate molecule. Another unique aspect of MonCI is that it acts on a substrate that is covalently tethered to a carrier protein. This modality is expected to confer the needed substrate specificity, presumably compensating for the fact that premonensin A is unable to establish extensive specific interactions with MonCI.

Phylogenetic analysis placed MonCI in a distinct clade from the other class A flavin-dependent monooxygenases (Supplementary Figs. 22 and 23). This clade contains epoxidases that are involved in the biosynthesis of salinomycin, tetronomycin, maduramicin, nigericin, and nanchangmycin, which are all polyether polyketide natural products produced by actinomycetes. While none of these epoxidases have been structurally characterized so far, we predict that they, like MonCI, contain an extra-large substrate-binding pocket and an active site that is surrounded by hydrophobic amino acids. Interestingly, this clade of polyether-producing monooxygenases shares the same main branch with Bmp2, the halogenase which installs four bromine atoms on a proline-derived carboxylate substrate that is tethered to a carrier protein^33^. MonCI and Bmp2, which use carrier-bound substrates, appear to have evolved separately from those that act on a free substrate such as the extensively studied 4-hydroxybenzoate hydroxylase. Our work adds another unique chemical transformation to the long list of enzymological functions performed by the superfamily of flavin-dependent monooxygenases.

## Methods

### MonCI and MonACPX cloning

MonCI gene from *Streptomyces cinnamonensis* was encoded into the pCold vector (Takara Bio USA) and transformed into *E. coli* BL21-AI. Gene fragments of MonACPX were synthesized and inserted into pET-28a (+) vector. Then PCR experiments were performed, and the genes of the control ACP were inserted into a plasmid containing an N-terminal SUMO-tag. All the mutagenesis experiments were done using overlap PCR. Sequencing was done for all the MonCI and MonACPX to confirm the accuracy of the cloning.

### MonCI expression and purification

Protein expression was induced using 100 µM isopropyl-ß-D-galactoside and 2 mg ml^−1^ l-arabinose and incubated for 20 h at 15 °C. Cells were harvested by centrifugation, resuspended in lysis buffer (50 mM sodium phosphate, pH 7.4, 300 mM sodium chloride, 40 mM imidazole, 10% glycerol) and lysed by sonication. After centrifugation at 18,000 × *g* for 40 min, the cleared supernatant was loaded onto a HisTrap column (GE Healthcare) and washed with wash buffer (50 mM sodium phosphate, pH 7.4, 300 mM sodium chloride, 70 mM imidazole, 10% glycerol). MonCI was eluted with a wash buffer supplemented with 200 mM imidazole. Fractions containing MonCI were diluted with loading buffer (20 mM Tris pH 8.5, 10% glycerol) and subjected to anion exchange and size-exclusion chromatography using a HiTrapQ (GE Healthcare) and a Superdex 75 10/300 GL column (GE Healthcare), respectively. The protein solution was concentrated to 6.2 mg ml^−1^ and stored at −80 °C. The overall yield was about 1.2 mg of MonCI per liter of culture and the protein purity was >95% as judged by polyacrylamide gel electrophoresis.

### MonACPX and DEBS ACP2 expression and purification

The wild-type MonACPX was induced using 100 µM isopropyl-ß-d-galactoside and incubated for 16–18 h at 18 °C. Cells were harvested by centrifugation, resuspended in lysis buffer (50 mM sodium phosphate, pH 7.4, 300 mM sodium chloride, 40 mM imidazole, 10% glycerol) and lysed by sonication. After centrifugation at 13,000 × *g* for 45 min, the cleared supernatant was loaded onto a HisTrap column (GE Healthcare) and washed with wash buffer (50 mM sodium phosphate, pH 7.4, 300 mM sodium chloride, 70 mM imidazole, 10% glycerol). MonACPX was eluted with a wash buffer supplemented with 250 mM imidazole. Purified MonACPX were incubated with buffer containing 20 mM Tris, pH 8.5, 150 mM sodium chloride, 2.5 mM calcium chloride and 100 U/ml thrombin for 20 h at room temperature to cleave the N-terminal His tag. The resulted MonACPX without the His-tag was further purified by size-exclusion chromatography was performed using a Superdex 75 Increase 10/300 GL column with the buffer containing 20 mM Tris, pH 7.5, 150 mM sodium chloride and 10% glycerol. The protein solution was concentrated to 11.26 mg ml^−1^ and stored at −80 °C.

MonACPX mutant and DEBS ACP2 expression was induced using 100 µM isopropyl-ß-d-galactoside and incubated for 16–18 h at 18 °C. Cells were harvested by centrifugation, resuspended in lysis buffer (50 mM sodium phosphate, pH 7.4, 300 mM sodium chloride, 40 mM imidazole, 10% glycerol) and lysed by sonication. After centrifugation at 13,000×*g* for 45 min, the cleared supernatant was loaded onto a HisTrap column (GE Healthcare) and washed with wash buffer (50 mM sodium phosphate, pH 7.4, 300 mM sodium chloride, 70 mM imidazole, 10% glycerol). MonACPX mutants were eluted with a wash buffer supplemented with 250 mM imidazole. The SUMO-tag in MonACPX and DEBS ACP2 were cleaved by ulp1 and then the proteins were purified by passing through the HisTrap column (GE Healthcare) again. The proteins were further purified by anion exchange using an HiTrap Q-HP column (GE Healthcare) with buffer A as 20 mM Tris, pH 7.5, 150 mM sodium chloride, 10% glycerol and bufffer B as buffer A supplemented 1 M sodium chloride. The proteins were finally purified by size-exclusion chromatography using a Superdex 75 Increase 10/300 GL column with the buffer containing 20 mM Tris, pH 7.5, 150 mM sodium chloride and 10% glycerol. MonACPX solutions was concentrated to 11.3 mg ml^−1^ and stored at −80 °C. DEBS ACP2 solution was concentrated to 8.9 mg ml^−1^ and stored at −80 °C.

### Glucose dehydrogenase expression and purification

pET-28a (+) vector carrying the *dhg* gene of *Priestia megaterium* was introduced into the *Escherichia coli* BL21(DE3) strain. The culture was grown in Luria Broth medium to OD_600nm_ of 0.6 and expression of N-terminal His-tagged glucose dehydrogenase was induced by 100 µM isopropyl-ß-d-galactoside. The culture was incubated for another 20 h at 18 °C.

Cells were harvested by centrifugation, resuspended in lysis buffer (50 mM sodium phosphate, pH 7.5, 300 mM sodium chloride, 10% glycerol) and lysed by sonication. After centrifugation at 13,000×*g* for 40 min, the cleared supernatant was loaded onto a HisTrap column (GE Healthcare) and washed with wash buffer (50 mM sodium phosphate, pH 7.5, 300 mM sodium chloride, 10% glycerol). Glucose dehydrogenase was eluted with a wash buffer supplemented with 250 mM imidazole. Fractions containing glucose dehydrogenase pooled and exchanged into a buffer containing 20 mM Tris, pH 7.5, 150 mM NaCl, 10% glycerol. The sample was concentrated to 10 mg ml^−1^ for storage.

### X-ray crystal structure determination

MonCI crystals were obtained from a 1:1 mixture of protein solution (6.2 mg ml^−1^ in 20 mM Tris pH 7.5, 150 mM sodium chloride, 10% glycerol) and reservoir solution (0.1 M Bis Tris pH 6.5, 0.1 M MOPS pH 6.8, 28% PEG2000mme, 4% glycerol) using the sitting drop vapor diffusion method. Protein crystals were transferred to a cryoprotectant solution comprised of the crystallization buffer supplemented with 20% glycerol prior to freezing. X-ray diffraction data from a native MonCI crystal and bromine soaked MonCI crystal were collected at beamline 12-2 of the Stanford Synchrotron Radiation Lightsource (SSRL). X-ray diffraction data were indexed, integrated, and scaled using the program autoXDS^63^, and the phase was solved by multi-wavelength anomalous diffraction using the program SHELX^64^. Initial MonCI model was generated using the program Buccaneer^65^. Iterative structure refinement and model building were carried out using the programs REFMAC^66^, Phenix_Refine^67^, and Coot^54^.

### Synthesis of farnesyl 4-hydroxybenzoate

Farnesol (1 g, 4.49 mmol) and p-hydroxybenzoic acid (0.9 g, 6.52 mmol) were dissolved in 60 ml of CHCl_2._ 4-dimethylaminopyridine (DMAP) (0.08 g, 0.65 mmol) and N,N’-dicyclohexylcarbodiimide (DCC) (1.15 g, 5.57 mmol) were slowly added at ice temperature. The mixture was stirred vigorously overnight at room temperature. Reaction was quenched using NaCl (3 ml std. sln). The organic layer was separated, and the aqueous phase was extracted with CHCl_2_ (10 mL x3). The combined organic layers were washed with NaHCO_3_ (80 mL), dried over Na_2_SO_4_ and the solvent was removed under reduced pressure. Flash chromatography (silica gel, 1:3 EtOAc—Petroleum ether) provided the product (0.48 g 31% yield). The final product was evaluated by nuclear magnetic resonance (NMR) (JOEL-ZETA 400 MHz and Bruker 400 MHz spectrometers). For mass spectrometry, 50 µg mL^-1^silver nitrate was added to the sample for better ionization. ^1^H NMR spectra were referenced internally to the residual proton resonance in CDCl_3_ (δ 1H = 7.26 ppm) as the internal standard. Chemical shifts (δ) were reported as part per million (ppm) in δ scale downfield from TMS. 1H NMR data were recorded as follows: multiplicity (s = singlet, d = doublet, t = triplet, m = multiplet or unresolved, coupling constant in Hz, integration).

^1^H NMR (400 MHz, CDCl_3_) δ 7.95 (d, *J* = 6.8 Hz, 2H), 6.85 (d, *J* = 18.3 Hz, 2H), 5.44 (t, *J* = 25.6 Hz, 1H), 5.10 (d, *J* = 15.3 Hz, 2H), 4.81 (d, 2H), 2.06 (m, 8H), 1.68 (s, 6H), 1.59 (s, 6H). ^13^C NMR (100 MHz, CDCl_3_) δ 167.29, 160.66, 142.73, 140.51, 135.66, 131.91, 124.32, 123.84, 123.12, 118.67, 115.26, 61.98, 44.30, 39.62, 31.99, 26.85, 25.65, 23.44, 17.55, 16.04. HRMS [M+Ag]+ = 449.1238 m/z. (Theoretical [M+Ag]+ for C_22_H_30_O_3_ = 449.1240.)

### Synthesis of 17,18-epoxy-farnesyl 4-hydroxybenzoate

Meta-chloroperoxybenzoic acid (0.268 g, 1.5 mmol) was dissolved in CH_2_Cl_2_ and added to FHB (0.3 g 0.88 mmol) in batches at 0 °C and left for 1 h. The reaction was quenched with 1 M NaOH and then extracted with ethyl acetate and washed with saturated NaHCO_3_. After drying with Na_2_SO_4_, the mixture was evaporated and separated by flash chromatography (silica gel,1:6 EtOAc—Petroleum ether), which provided the product as a clear, colorless liquid (0.09 g, 29% yield). The final product was evaluated by NMR (JOEL-ZETA 400 MHz and Bruker 400 MHz spectrometers) and LC-HRMS (Agilent X500R QTOF, ESI, resolution 0.0001 *m/z*).

^1^H NMR (400 MHz, CDCl_3_) δ 7.95 (d, *J* = 8.8 Hz, 2H), 6.85 (d, *J* = 8.8 Hz, 2H), 5.49 (d, *J* = 13.1 Hz, 1H), 5.24–5.05 (m, 1H), 4.80 (dd, *J* = 6.9, 2.5 Hz, 2H), 2.70–2.75 (m, 1H), 1.66 (m, 12H), 1.33–1.20 (m, 2H), 0.84 (m, 6H). ^13^C NMR (100 MHz, CDCl_3_) δ 167.29, 160.66, 142.73, 140.51, 135.66, 124.32, 123.84, 118.67, 115.26, 64.76, 61.98, 58.90, 44.30, 39.62, 31.99, 26.85, 25.65, 23.44, 17.55, 16.04. HRMS [M + H]^+^ = 359.2223 *m/z* (theoretical [M + H]^+^ for C_22_H_31_O_4_ = 359.2216).

### In vitro enzyme activity assay

The reaction mixture contained either (1) 50 mM Tris, pH 8.0, 160 µM FHB, 15 µM MonCI, 4 mM NADH, 80 µM FAD, 15 µM Fre, 300 mM NaCl, and 10% glycerol, or (2) 50 mM Tris, pH 8.0, 160 µM FHB, 15 µM MonCI, 15 µM MonCI, 80 µM FAD, 300 mM NaCl, 15 µM GDH, 80 mM D-glucose, 4 mM NAD^+^, and 10% glycerol. The reaction mixture was incubated for 6 h at 30 °C. Ethyl acetate was used to extract the unreacted substrate and product, and then vacuum evaporated to obtain samples for LC-MS analysis. Samples were dissolved in methanol and centrifuged at high speed for 10 min prior to LC-MS analysis. Large scale enzymatic assay was performed in 20 ml reaction system with 50 mM Tris, pH 8.0 buffer, 160 µM FHB, 15 µM MonCI, 4 mM NADH, 15 µM Fre, 300 mM NaCl, and 10% glycerol for 6 h at 30 °C. After the reaction, the enzymatic catalytic product was extracted by ethyl acetate, evaporated, dissolved by methanol and purified by HPLC (EClassical UV3100 P3500, Column: UniSil 10–120 C18 Ultra 10 µm 21.2 × 250 mm).

The control experiments of the enzymatic assay without MonCI or cofactor NADH were performed with other conditions the same for the reactions. The products were extracted by ethyl acetate, vacuum evaporated, dissolved in methanol and and centrifuged at high speed for 10 min prior to LC-MS analysis. LC-MS (Agilent 6460 Triple Quad LC/MS, resolution 0.01 *m/z*) was applied with an Agilent ZORBAX Eclipse XDB-C18 column at a flow rate of 0.2 mL/min and column temperature of 30 °C. Buffer A was 99.9% H_2_O and 0.1% formic acid, and buffer B was 99.9% methanol and 0.1% formic acid. Mass spectra were collected in positive ion mode 100–1000 *m/z*. The program was set as: 60% B at time 0–0.5 min, 60% B and 60–95%B from 0.5 to 6 min, 95–60% B from 6 to 8 min, 60% B from 8 to 9 min. For FHB epoxidation products, peak 365.2093+ *m/z*, 381.2042+ *m/z*, 397.1991+ *m/z* were selected for analysis.

To quantify the amount of the enzymatic catalytic product, HPLC (Shimadzu LC-2010A HT) analysis was performed. Buffer A was H_2_O and buffer B was methanol. With the gradient of 40–5% A, the monoepoxide FHB product was eluted at a retention volume of 10 ml and collected. The product was further analyzed by HRMS (Agilent X500R QTOF, ESI, resolution 0.0001 *m/z*) in a negative mode. The observed *m/z* was 357.2072 matching the theoretical Monoepoxide [M-H]^-^ = 357.2060 *m/z*.

High-resolution LC-MS (Agilent X500R QTOF, ESI, resolution 0.0001 *m/z*) was employed to determine the mass of the substrate and products from the MonCI catalytic reactions. The products of reaction (1) and (2) were processed the same as the above methods and applied to column Kinetex 2.6um C18 100 with a flow rate of 0.3 ml/min and column temperature of 30 °C.The program was set as: 5% B at time 0–1 min 5–95% B from 1 to 3 min, 95% B from 3 to 8 min, and 95% B–5% B from 8 to 8.1 min. For the enzymatic reaction carried out under condition (1), the detected masses were 341.2119 *m/z* for FHB, 357.2072 *m/z* for monoepoxide, and 373.2019 *m/z* for bisepoxide. For the enzymatic reaction carried out under condition (2), the detected masses were 343.2269 *m/z* for FHB, 359.2221 *m/z* for monoepoxide, 375.2177 *m/z* for bisepoxide, and 391.2128 *m/z* for triepoxide. These mass spectra were collected in positive ion mode.

### Colorimetric assay for epoxides

66 µL NBP (6wt/vol%, 19.5 vol% 1-butanol, 80 vol% propylene glycol, 0.5 vol% acetic acid) was added to 100 µL 160 µM purified enzymatic catalytic product, 160 µM synthetic 17,18-epoxy-4-hydroxybenzoate standard in methanol, or methanol. Then the mixture was transferred to three centrifuge tubes and heated in an 80 °C oven for 2.5 h. Then the tubes were taken out and put on ice for 10 min and 66 µL triethylamine acetone solution (1:1 mixture) was added immediately to the mixture. After high-speed centrifugation, the supernatants were scanned with an ultraviolet spectrophotometer (UV1800 PC) for an absorption peak at a wavelength between 200 and 700 nm, and a spectrogram was drawn.

### Homology modeling

Sequence alignment of ACPs was performed by CLUSTALW^68^ and ESPript server^69^, which identified the ACP domain from module 5 of MLSA1 (PDB ID: 6H0J) as the best template structure to use in homology modeling of MonACPX. The homology structure of MonCI was generated using the program MODELLER^70^. Evaluation of structural models was performed by calculation of z-DOPE score with MODELLER program and the best MonACPX model was selected with comparing the score for all residues. Visualization and image processing were performed by Pymol and Maestro 12.2 (Schrödinger, LLC, Portland, OR) program.

### Molecular docking

Structures of the MonACPX-MonCI complex were predicted using the HADDOCK2.2 webserver^50,71^. Docking was performed using the MonCl crystal structure and the homology model of *apo*-MonACPX. The HADDOCK2.2 webserver’s default input settings were used, and passive residues were assigned automatically around the active residues. Active residues for MonCl were those located near the substrate-binding pocket entrance (side entrance): Asp377, Asp378, Ile381, Thr398, Asp399, Pro400, Arg401, Leu402, Ile403, Gly404, Val405, Asp406, Gln409, Arg412, Phe413, Pro440, Gln441, Ala442, Glu443, Gly445, Ser446, Asn447, Arg448, and Leu450. Two docking runs were performed using different active residues for ACP. First run was performed defining a narrower (Thr37, Gln38, Ser60, Leu61, Leu64, Glu65, Lys68, Thr69, Met80) and second run a wider (Thr37, Gln38, Ala39, Gly40, Asn59, Ser60, Leu61, Thr62, Leu64, Glu65, Thr67, Lys68, Thr69, Met80) selection of active residues surrounding Ser60 of MonACPX, the attachment site for the 4´-phosphopantetheine group. Among the docked structures that were generated, we eliminated those in which Ser60 was not aligned with the side entrance and then chose the solution that had the best HADDOCK score (−86.2 ± 3.6) as the final model MonACPX-MonCI.

### Molecular dynamics simulation

Each simulated system contained MonCI, FAD, and except for one system, premonensin A. The MonCI and FAD coordinates were obtained from chain A of the crystal structure (PDB ID: 8T3P). 481 water molecules that are in the crystal structure and are associated with chain A were also included in simulations. In all cases, hydrogen atoms were added using the VMD *psfgen* plugin^72^. For simulations which included premonensin A, the substrate was manually positioned within the channel with the substrate orientation varying by system. The initial substrate position was an approximate binding pose with the reactive double bond placed ~5 Å from and perpendicular to the O-H bond of the reactive −OOH group of FAD. The systems were solvated and ionized using the VMD *solvate* and *ionize* plugins^72^. Final systems contained approximately 78,000 atoms, with a salt concentration of 150 mM NaCl to mimic physiological conditions.

Interactions for simulated molecules were defined using the CHARMM36 force-field parameters^73^ and the TIP3P water model, with the CHARMM general force field (Cgenff)^74–77^ used to model the substrate. MD simulations were performed in the NpT ensemble using the NAMD2.13 package^78^. Temperature and pressure remained constant at 310 K and 1.01325 bar, respectively. The Langevin constant was γ_Lang_ = 1 ps^−1^. A simulation timestep of 2 fs was used, with evaluation of van der Waals interactions at every timestep and evaluation of long-range Coulomb interactions performed every two timesteps using the particle-mesh Ewald method^79^.

All systems were initially minimized for 2,000 steps, after which water and ions were equilibrated for 1 ns while the protein-FAD-substrate complex was restrained using harmonic forces with a spring constant of 1 kcal/(mol Å^2^). A further 1 ns of equilibration was performed with only the heavy atoms of the protein-FAD-substrate complex restrained, followed by 20 ns of equilibration in which disordered coil protein residues were not restrained. A final 20 ns equilibration run was performed with harmonic restraints present only on the substrate atoms constituting the double bond nearest FAD. Production runs, with all restraints released, were performed for 200 ns. The analyses were performed during the last 100 ns of the production runs.

Images of simulated systems were prepared with VMD, with secondary structure information obtained using STRIDE^80^. RMSDs were calculated using the VMD RMSD trajectory tool. The channel residues include MonCl residues 55, 56, 57, 58, 59, 60, 88, 92, 94, 95, 96, 97, 98, 99, 101, 112, 206, 208, 209, 210, 226, 227, 228, 229, 230, 232, 239, 240, 241, 242, 243, 244, 253, 254, 255, 256, 273, 277, 282, 329, 330, 331, 332, 333, 334, 336, 382, 385, 386, 387, 390, 414, 415, 417, 418, 421, 422, 431, 435.

### Isothermal titration calorimetry

All protein samples were buffer exchanged into buffer 100 mM phosphate, pH 8.0. All the samples were degassed by centrifugation at 15000 rpm for 10 min. When using wild-type MonACPX to titrate wild-type MonCI, wild-type MonCI (60 μM) was in the cell while wild-type MonACPX (1.2 mM) was in the syringe. When using control ACP to titrate wild-type MonCI, wild-type MonCI (100 μM) was in the cell while control ACP (1.0 mM) was in the syringe. The concentrations of MonCI were determined by UV_280nm_ method. The concentrations of MonACPX, its mutants and control ACP were determined by Bradford method. ITC titrations were using the MicroCal ITC200 system at 25 °C. There were twenty titrations for each experiment (the injection is 0.5 μL for the first titration and 2 μL for the rest 19 titrations). The ITC data was integrated with a “one site” model. All titration experiments were performed twice. The software Origin 7.0 (OriginLab) was used for data analysis.

### Ultraviolet–visible spectrophotometry

The flavin absorption spectra of purified MonCI were analysed with a Jinghua UV1800 PC spectrophotometry. Untreated MonCI (as isolated from *E. coli*) was scanned to obtain the MonCI-Flox[O] spectrum. After incubating MonCI with FHB for 12 h, the sample was buffer exchanged to the enzymatic reaction buffer to remove the cofactors as well as the substrate and product and was scanned to obtain the spectrum of MonCI-Flox.

### Phylogenetic analysis of group A FMO

The phylogenetic tree was calculated based on the amino acid sequence alignment of biochemically characterized enzymes’ sequences by using the ClustalW method in MEGA11 as reported by Adrie H. Westphal et al.^81^ Subsequently, the tree was generated using a neighbor-joining method, while evolutionary distances were calculated using a JTT matrix-based method. A bootstrap consensus tree inferred from 1000 replicates was used to represent the evolutionary history of the analyzed protein sequences. Next to the branches is shown the percentage of replicate trees in which related sequences clustered together in a statistical test (1000 replicates).

### Reporting summary

Further information on research design is available in the Nature Portfolio Reporting Summary linked to this article.

### Supplementary information

Supplementary Information

Reporting Summary

### Source data

Source data file

## Data Availability

The atomic coordinates and structure factors generated in this study have been deposited in the RCSB Protein Data Bank under accession code 8T3P. Source data are provided with this paper.
