# Supplementary material for: Triepoxide formation by a flavin-dependent monooxygenase in monensin biosynthesis
Source: Nat Commun. 2023 Oct 7;14:6273. doi: 10.1038/s41467-023-41889-0 (PMC10560226; doi:10.1038/s41467-023-41889-0)
Supplement: Supplementary file 1 — Supplementary Information [file 41467_2023_41889_MOESM1_ESM.pdf]

Supplementary Information for

Triepoxide formation by a flavin-dependent monooxygenase in monensin biosynthesis

Qian Wang[1,4], Ning Liu[2,4], Yaming Deng[2,4], Yuze Guan[2], Hongli Xiao[2], Tara A. Nitka[1], Hui Yang[2], Anju Yadav[1], Lela Vukovic[1], Irimpan I. Mathews[3], Xi Chen[2,*] and Chu-Young Kim[1,5,*]

[1]Department of Chemistry and Biochemistry, The University of Texas at El Paso, 500 West University Avenue, El Paso, TX 79968, USA.

[2]Key Laboratory of Synthetic and Natural Functional Molecular Chemistry of Ministry of Education, College of Chemistry and Materials Science, Northwest University, Xi'an, 710127, China.

[3]Stanford Synchrotron Radiation Lightsource, SLAC National Accelerator Laboratory, 2575 Sand Hill Road, Menlo Park, CA 95124, USA.

[4]These authors contributed equally: Qian Wang, Ning Liu, Yaming Deng

[5]Current address: Department of Biochemistry, University of Illinois Urbana-Champaign, Urbana, IL 61801, USA.

*Corresponding author: Chu-Young Kim (chuyoung@illinois.edu), Xi Chen (xchen@nwu.edu.cn)

Supplementary Table 1. Data collection and refinement statistics.

	MonCI	Br derivative	Br derivative	Br derivative
**Data collection**				
Space group	*P*21	*P*21	*P*21	*P*21
Cell dimensions				
a, *b*, *c* (Å)	61.2, 52.4, 141.6			
α, β, γ (°)	90.0, 93.6, 90.0			
	Native	*Peak*	*Inflection*	*Remote*
Wavelength (Å)	0.9795	0.9200	0.9392	0.9795
Resolution (Å)	1.90 (1.95-1.90)	2.25 (2.31-2.25)	2.25 (2.31-2.25)	2.1 (2.15-2.10)
R_{sym} (%)	12.3 (131)	21.5 (161)	22.3 (166)	18.5 (141)
I/σ (I)	13.6 (1.63)	15.4 (1.31)	12.4 (1.44)	13.5 (1.62)
Completeness (%)	96.4 (95.8)	97.5 (98.3)	97.5 (98.4)	97.6 (98.)
Redundancy	8.0 (8.1)	33.9 (16.4)	24.3 (24.6)	24.3 (24.6)
$CC_{1/2}$	0.999 (0.658)			
**Refinement statistics**				
Resolution limit (Å)	39.7-1.9 (1.97-1.90)			
No. of reflections	73,562 (3,741)			
R_{work} / R_{free} (%)	18.1/22.2			
**Number of atoms**				
Protein	7,180			
Ligand	106			
Ion	2			
Water	721			
**B-factors (Å^2)**				
Protein	35.0			
Ligand	27.4			
Ion	42.7			
Water	35.3			
**R.m.s deviations**				
Bond lengths (Å)	0.015			
Bond angles (°)	1.185			

Values in parentheses are for the highest resolution shell.
$R_{sym} = \Sigma \mid I\text{avg} - I\text{i} \mid / \Sigma I\text{i}$, where Ii is the observed intensity and *I*avg is the average intensity of observations of symmetry-related reflections.
$R_{work} = \Sigma \mid F\text{p} - F\text{p(calc.)} \mid / \Sigma F\text{p}$, where *F*p and *F*p(calc.) are observed and calculated structure factors; R_{free} is calculated with 5% of the data.

Supplementary Table 2. Sequence analysis of MonCI and its homologs. The proteins are listed in decreasing order of sequence identity to MonCI: NanO 59.8%, SalC 52.6%, TmnC 49.5%, Lsd18 47.8%, Bmp2 14.8%, PHBH 14.8%, SQLE 14.1%. The two conserved sequences (Residues 47-64 and 327-340 in MonCI), which are found only in polyether producing monooxygenases, are highlighted in red and yellow. MonCI (*Streptomyces cinnamonensis*), NaO (*Streptomyces nanchangesis*), TmnC (*Streptomyces sp.* NRRL 11266), SalC (*Streptomyces albus* subsp. albus), Lsd18 (*Streptomyces lasaliensis*), Bmp2 (*Pseudoalteromonas sp.* PS5), PHBH (*Pseudomonas fluorescens*), and SQLE (*Homo sapiens*).

```
MonCI      ------------------------------------------------------------      0
NanO       ------------------------------------------------------------      0
SalC       ------------------------------------------------------------      0
TmnC       ------------------------------------------------------------      0
Lsd18      ------------------------------------------------------------      0
Bmp2       ------------------------------------------------------------      0
PHBH       ------------------------------------------------------------      0
SQLE       MWTFLGIATFTYFYKKFGDFITLANREVLLCVLVFLSLGLVLSYRCRHRNGGLLGRQQSG      60

MonCI      ---------------------------------------------------------MTT      3
NanO       -----------------------------------------------------------M      1
SalC       ------------------------------------------------MPAADGRVTHLM      12
TmnC       ----------------------------------------------------MAEATRGP      8
Lsd18      -----------------------------------------------------------M      1
Bmp2       ------------------------------------------------------GSHMNG      6
PHBH       ------------------------------------------------------------      0
SQLE       SQFALFSDILSGLPFIGFFWAKSPPESENKEQLEARRRRKGTNISETSLIGTAACTSTSS      120

MonCI      TRPAHAVVLGASMAGTLAAHVLARHVDAVTVVERDA-LPEEP--QHRKGVPQARHAHLLW      60
NanO       TTPTRAVVLGGGWAGMLTAHVLARHLESVTVVERDI-LPDGP--HHRKGQPQARHVHVLW      58
SalC       TGETHAVVLGGGLTGMLTSAVLARHLDKVTVIERDV-LPEGP--ELRKGVPQARHAHLLW      69
TmnC       NTRVHGVVLGGGLAGVLAARALRDHVDHVTVVERDT-YPDLT--EPRKGVPQGRHAHILW      65
Lsd18      TNTRSAVVLGGGMAGMLVSSMLARHVGSVTVIDRDA-FPAGP--DLRKGVPQARHAHILW      58
Bmp2       FTHYDVVIIGSGPAGSLCGIECRKKGLSVLCIEKE-QFPRFHIGESLTG----NAGQIIR      61
PHBH       -MKTQVAIIGAGPSGLLLGQLLHKAGIDNVILERQ--TPDYVLGRIRAGVLEQGMVDLLR      57
SQLE       QNDPEVIIVGAGVLGSALAAVLSRDGRKVTVIERDLKEPDRIVGEFLQP----GGYHVLK      176
                  ::*..  *   .            ::::   *                 .::

MonCI      SNGARLIEEMLPGTTDRLLAAGARRL-G--------FPEDLVTLTGQGWQHRFPATQFAL      111
NanO       SSGAGIVENLLPGTAERLLAAGARRI-G--------FQSDLVTLTAWGWQYRFPATAYAM      109
SalC       SGGARAIDSVLPGTVKQLIAEGAHRL-Y--------LPRDVVWLTPHGWQHRFSGSQFMV      120
TmnC       SGGAEAIEELLPGTLDRLRAAGAHRI-G--------VKEDMVLYSAYGWQHRFPGSHYAL      116
Lsd18      SGGARIVEELLPGTTDRLLGAGAHRI-G--------IPDGQVSYTAYGWQHRFPEAQFMI      109
Bmp2       DLG----------LAEDMDAAGFPDKPGVNVIGSLSKNEFFIPILAPTW-QVR-------      103
PHBH       EAG----------VDRRMARDGL-VHEGVEIAFAGQRRRIDLK-------RLSGGK----      95
SQLE       DLG----------LGDTVEGLDAQVVNGYMIHDQESKSEVQIPYPLSENNQVQSGR----      222
           . *              :   .                   :

MonCI      VASRPLLDLTVRQQALGADNITVRQRTEAVELTGSGGGSGGRVTG--VVVRDLDS--GRQ      167
NanO       MCTRPLLDWVVRDAILAGGRIEVEHGTEAVELAGD----RSRVTG--VRVRDAGG--GEP      161
SalC       TCSRALLDWVVRRQALAEPKITVRQETEVLGLLGG----AGQVTG--VRLRDR-S--GES      171
TmnC       TCSRPLLDRTVREAALDHPDTEVLTRTEAHGLLGD----RTSVTG--VRVRTS-D--GAT      167
Lsd18      ACSRALLDWTVREETLREERIALVEKTEVLALLGD----AGRVTG--VRVRDQES--GEE      161
Bmp2       ---RSDFDD-MIKRKAVEHGVEYKLGMV-TDVIKD----GEKVVG--ALYKADGV--EHQ      150
PHBH       T-----------------VTVYGQTEVTRDLMEA----R-EACGATTVYQAAEVRLHDL      132
SQLE       AFHHGRFIMSLRKAAMAEPNAKFIEGVV-LQLLEE----DDVVMG--VQYKDKET--GDI      273
                                          :          . *  .  :

MonCI      EQLEADLVIDATGRGSRL---------------KQWLAALGVPALEEDVVDAGVAYATRL      212
NanO       RLLEADLVVDATGRASRL---------------GHWLAALGLPAVEQDVVDAGIGYATRM      206
SalC       TELAADLVVDAGGRASAL---------------RRWLPELGLDQVEEDVVDSGIAYATRV      216
TmnC       RELPADIVVDATGRGSRL---------------RHWLTDLGLPPAAEESVDTGLTYATRV      212
Lsd18      REVPADLVVDTTGRGSPS---------------KRLLAELGLPAPEEEFVDSGMVYATRL      206
Bmp2       --VRSKVLVDASGQNTFLS------RKGVAGKRQIEFFSQQIASFAH--------YKGVE      194
PHBH       QGERPYVTFERDGERLRLDCDYIAGCDGFHGISRQSIPAERLKVFERVY---PFGWLGLL      189
SQLE       KELHAPLTVVADG---------------LFSKFRKSLVSNKVSVSSH--------FVGFL      310
                 : .   *                       :    :    .        :

MonCI      FKAPPGATTHFPAVNIAADDRVREPGRFGVVYPIE-----------GGRWLATLSCTRGA      261
NanO       FKAPEGADGNFPAVQVAADPLTRQPGRFGVVYPQE-----------GGRWLVTLTSTRGA      255
SalC       FKAPAPVAQGFPMVNIAAAPGLGKPGQNGALVPIE-----------DGKWLVTLAGTRGG      265
TmnC       FRAPAGAPGAFPVVSVYADHRSGEPGRNGLLLPIE-----------DGRWIITLSGTRGG      261
Lsd18      FRAPEAAATNFPLVSVHADHRAGRPGCNAVLMPIE-----------DGRWIVTVSGTRGG      255
Bmp2       -RDLPPFSTN------TTILYSKQYHWS-WIIPISPDTDSLGVVIPKDLYYKECKNPDDA      246
PHBH       -ADTPPVS--------HELIYANHPRGFALCSQRS---------ATRSRYYVQVPLTEKV      231
SQLE       MKNAPQFKAN------HAELILANPSPV-LIYQISSSETRVLVDI-------RGEMPRNL      356
                                  .          .
```

```
MonCI      QLPTHEDEFIPFAE-NLNHPILADLLRDAEPLTPVFGSRSGANRRLYPERLEQWPDGLLV      320
NanO       PLPTDEDEFTGYAK-VLRHSIVSELMSVAEPISPIFQSHSGANRRMYPERMPQWPEGLLI      314
SalC       EPPTDDDSFLDFAR-GLRHPVLADLLERAEPLGPVKGSRSTVNRRLYYDRVANWPDGLLV      324
TmnC       EPTADEERFATFAR-SLRDPIIADLIEAAEPLTPVRTTRSTLNRRMHLDRLADRPEGLVA      320
Lsd18      EPPADDEGFARFARDGVRHPLVGELIAKAQPLTSVERSRSTVNRRLHYDRLATWPEGLVV      315
Bmp2       IAWGM-DHISPEL----KRRF-----KNAERQGD---SQSMADFSYRIEP--FVGDGWMC      291
PHBH       EDWSD-ERFWTELKARLPAEV-----AEKLVTGPSLEKSIAPLRSFVVEP--MQHGRLFL      283
SQLE       REYMV-EKIYPQIPDHLKEPF-----LEATDNSH---LRSMPASFLPPSS--VKKRGVLL      405
                 : :           .                           .         .

MonCI      IGDSLTAFNPIYGHGMSSAARCATTIDREFER------------SVQEGT--GSARAGTR      366
NanO       LGDSLAAFNPVYGHGMSSAARAAEALDKELAR------------D-------GFGEGGTR      355
SalC       LGDALAAFNPVYGHGMSCSALSAKALDAELGR-----------------S--GLAPGMVQ      365
TmnC       LGDCVVSLNPIHGHGMSVAARSARALEACLSR------------A----G--GLKPGLAR      362
Lsd18      LGDAVAAFNPVYGHGMSAAAHSVLALRSQLGQ-----------------R--AFQPGLAR      356
Bmp2       IGDAHRFLDPIFSYGVSFAMKEGIRAAEAIAQVVAGQDWKAPFYAYRDWSNGG-------      344
PHBH       AGDAAHIVPPTGAKGLNLAASDVS------------TLYRLLLKAYREGRGELLERY-SA      330
SQLE       LGDAYNMRHPLTGGGMTVAFKDI-------------KLWRKLLKGIPDLYDDA-------      445
            **.     *  . *:. :

MonCI      ALQKAIGAAVD-DPWILAATKDIDYVNC---------RVSATDPRLIGVDTEQRLRFAEA      416
NanO       QVQRALSEVVD-DPWIMAGLNDIQYVNC---------RNLSSDPRLTGPDVAERLKFSDF      405
SalC       AVLQKVAKVVD-DPWLLATTQDICYPGT---------KVTAQDPRIAP-RGDQEQQFADL      414
TmnC       TAQQAIAAAAD-APWLLSASQDLCYPDN---------KAAVSDPRLTT-QAAQRQGFADM      411
Lsd18      AAQRAIAVAVD-DAWVLATSHDIGYPGC---------RTQTRDPRLTR-HAGERQRVTDL      405
Bmp2       ---QQIAADLIRYFWIYP----------------IFFGYQMQNPDLRDE---V----IRL      378
PHBH       ICLRRIWKAERFSWWMTSVLHRFPD-------------TDAFSQRIQQT---E---LEYY      371
SQLE       ----AIFEAKKSFYWARKTSHSFVVNILAQALYELFSATDDSLHQLRKA---C--FLYFK      496
                :        *                              :

MonCI      ITAASIRSPKASEIVTDVMSLNAPQAELGSNRFLMAMRADERLPELTAPPFLPEELAVVG      476
NanO       LSGKSIRSPKVCEVTTSVLSLNAPQKALGDSRFLSLLRTDTSHPKLVEPPFHPEELEMVG      465
SalC       LSTAALHDPVVSAAAMQVTALAAPVSSLESPHLVAALRKGKAHEPLTAPPFKDAELAVLD      474
TmnC       VTSASLVNERVCDALTAVTTLTAPLGSLETPEFLAAMRQ-PARPPLTAAPLKDAEAAVLR      470
Lsd18      VGLTATRNQVVNRAAVALNTLSAGMASMQDPAVMAAVRRGPEVPAPTEPPLRPDEVARLV      465
Bmp2       LG---------------------------------------GCCFDCEGWKAPAI--FR      396
PHBH       LGSEAGLAT------IAENYV--GLPYEEIE-----------------------------      394
SQLE       LGGECVAGP------VGLLSVLSPNPLVLIGHFFAVAIYAV-YFCFKSEPWITKPRALLS      549
           :

MonCI      LDAATISPTPTPTPTAAVRS-----      496
NanO       LKPSGIAAKGALG------------      478
SalC       GDAAGAAASA---------------      484
TmnC       AAK----------------------      473
Lsd18      SGAGVTA------------------      472
Bmp2       NAIEEYDRKQ------------MAS      409
PHBH       -------------------------      394
SQLE       SGAVLYKACSVIFPLIYSEMKYMVH      574
```

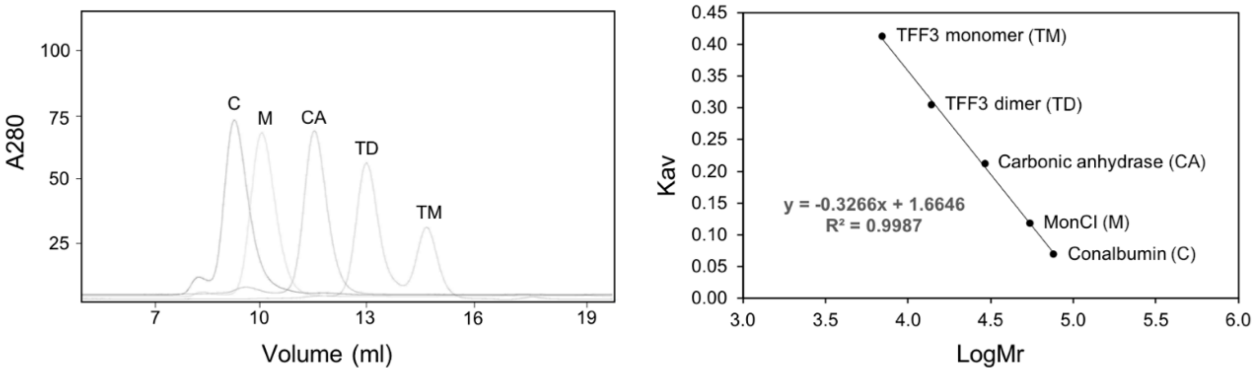

Supplementary Fig. 1 | Size exclusion chromatography analysis of MonCI. Superdex 75 10/300 GL column (GE Healthcare) was used. Theoretical molecular weight of MonCI monomer is 55.1 kDa and the experimentally determined molecular weight is 53.8 kDa. Human intestinal trefoil factor monomer (6.86 kDa), human intestinal trefoil factor dimer (13.7 kDa), carbonic anhydrase (29 kDa), and conalbumin (75 kDa) were used as molecular weight standards.

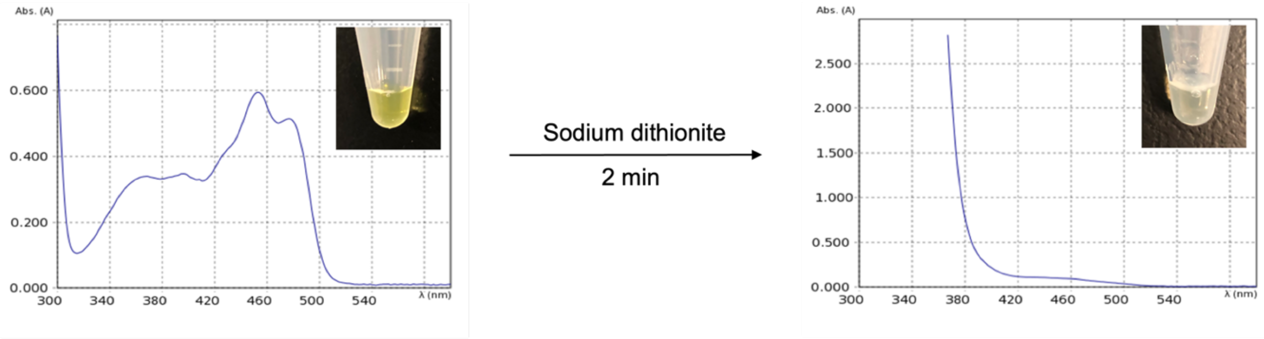

Supplementary Fig. 2 | Ultraviolet-visible spectrum of recombinant MonCI before and after addition of sodium dithionate. Solution containing MonCI appears yellow because it contains an oxidized flavin adenine dinucleotide coenzyme. Addition of sodium dithionite reduces flavin adenine dinucleotide, resulting in a colorless solution.

a

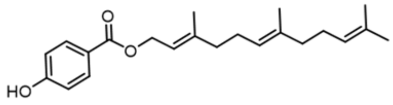

b

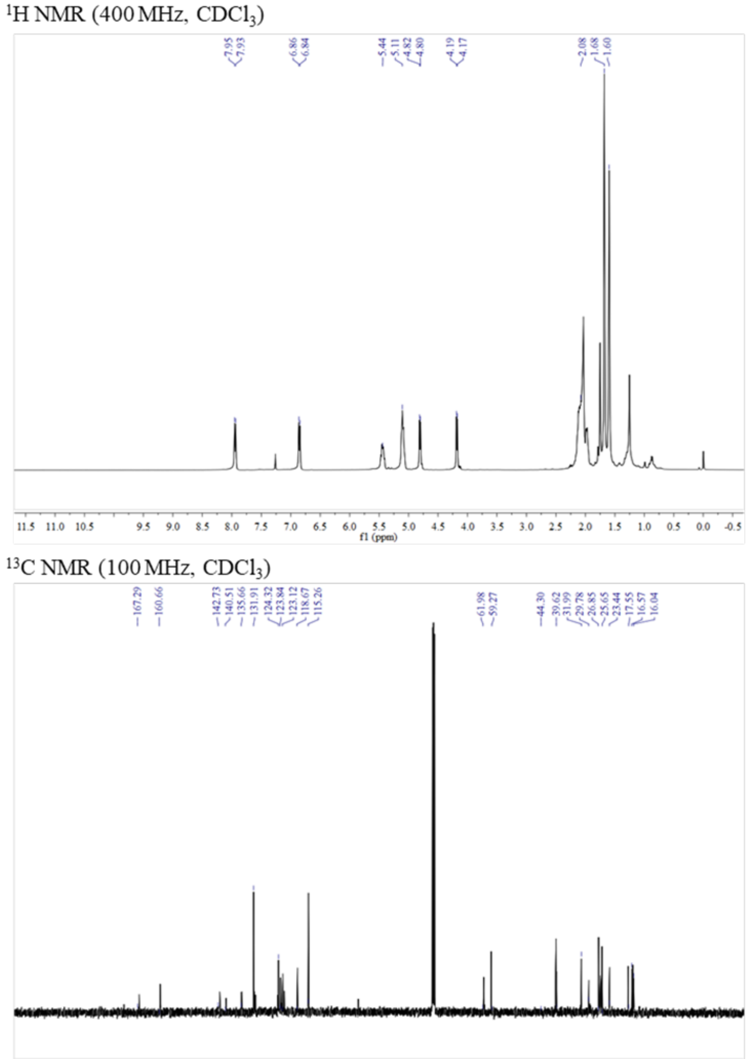

c

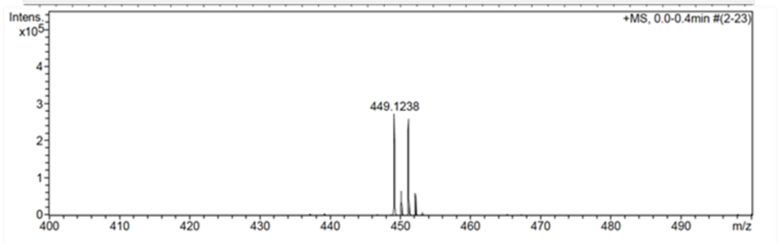

Supplementary Fig. 3 | Chemically synthesized farnesyl 4-hydroxybenzoate. a Chemical structure. **b** NMR spectrum. **c** Positive ion electrospray ionization mass spectrum. Theoretical $[M+Ag]^+$ = 449.1240 m/z.

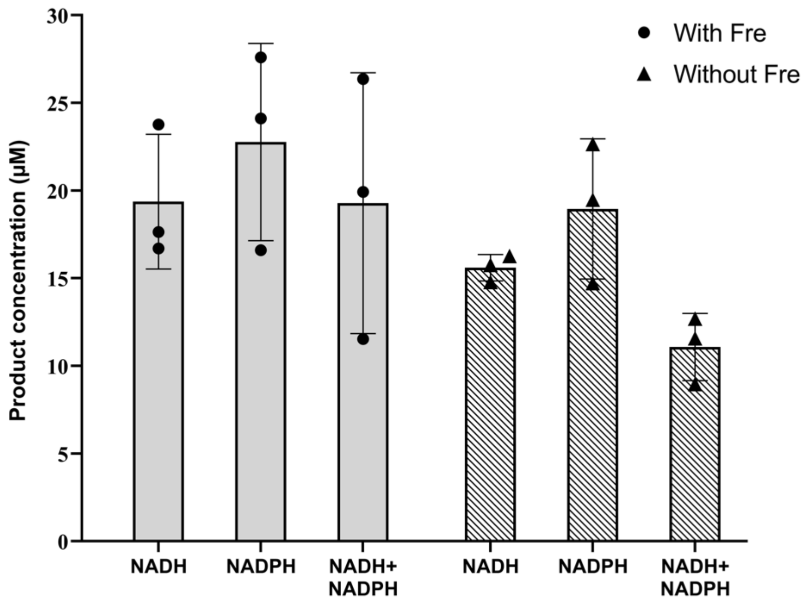

Supplementary Fig. 4 | Cofactor preference of MonCI and effect of Fre determined using HPLC. Data are presented as mean values ± SD (n = 3).

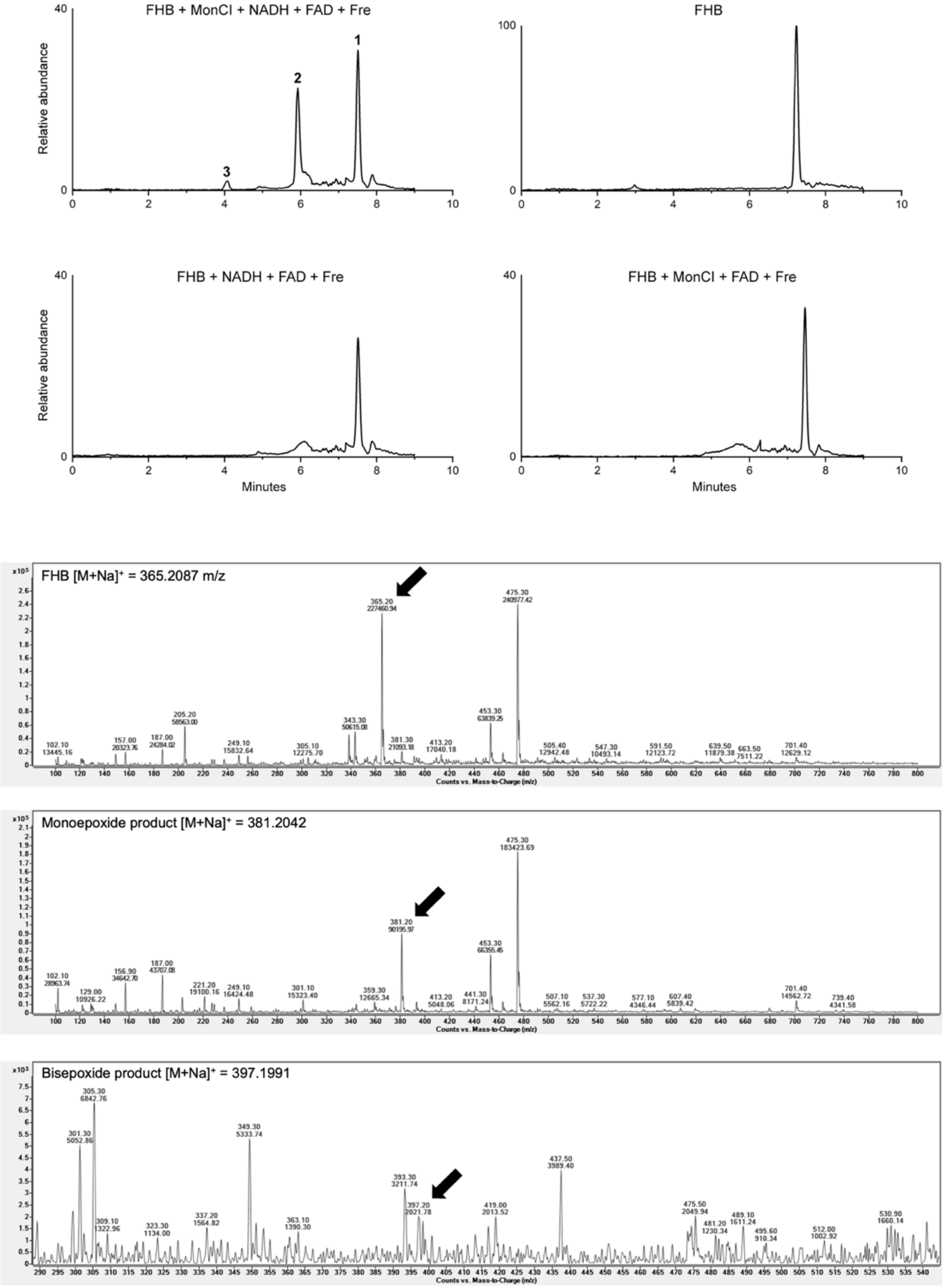

Supplementary Fig. 5 | LC-MS based MonCI activity assay. 1 is unreacted FHB, **2** is epoxy-FHB, and **3** is bisepoxy-FHB. Total ion current chromatogram is obtained by plotting the total ion current detected in each of a series of mass spectra recorded as a function of retention time. Mass spectra were collected in positive ion mode over 100-1,000 m/z. Source data are provided as a Source Data file.

a

b

c

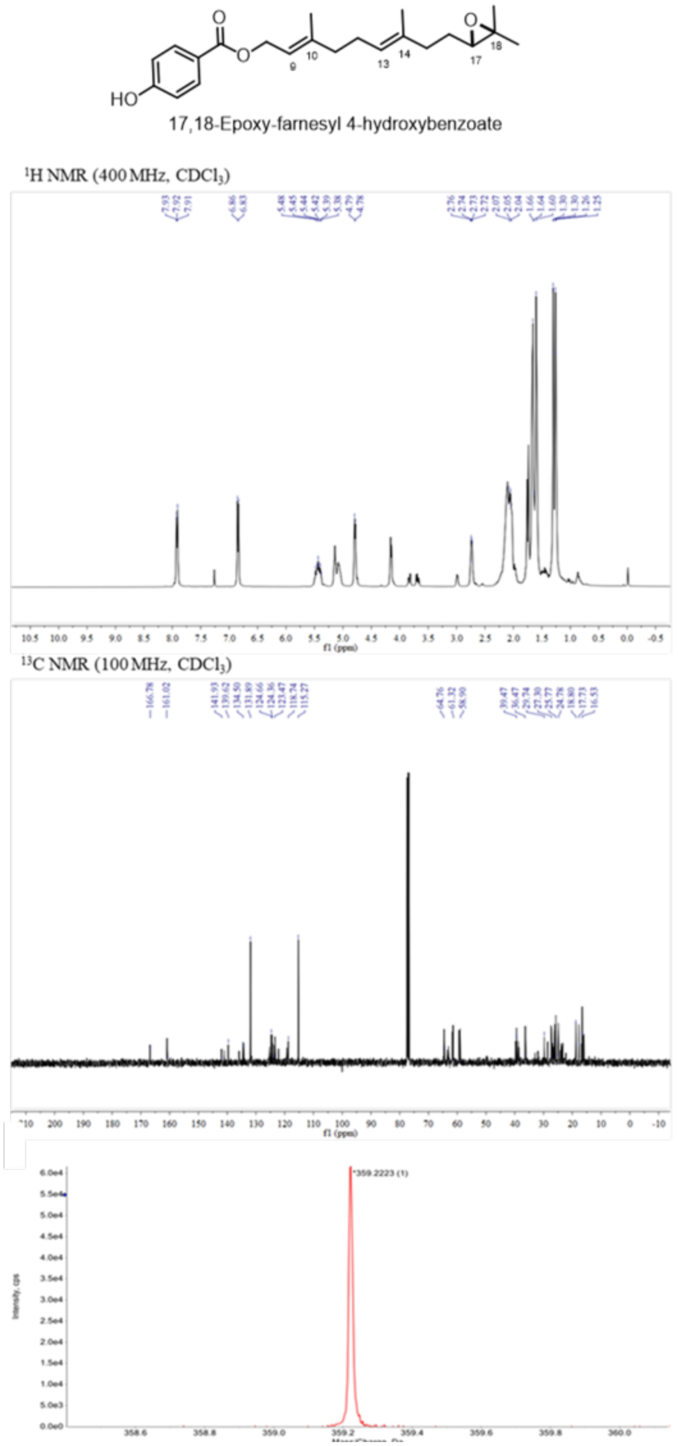

Supplementary Fig. 6 | Chemically synthesized 17,18-epoxy-farnesyl 4-hydroxybenzoate. a Chemical structure. **b** NMR spectrum. **c** Positive ion electrospray ionization mass spectrum. Theoretical $[M+H]^+$ = 359.2216 m/z.

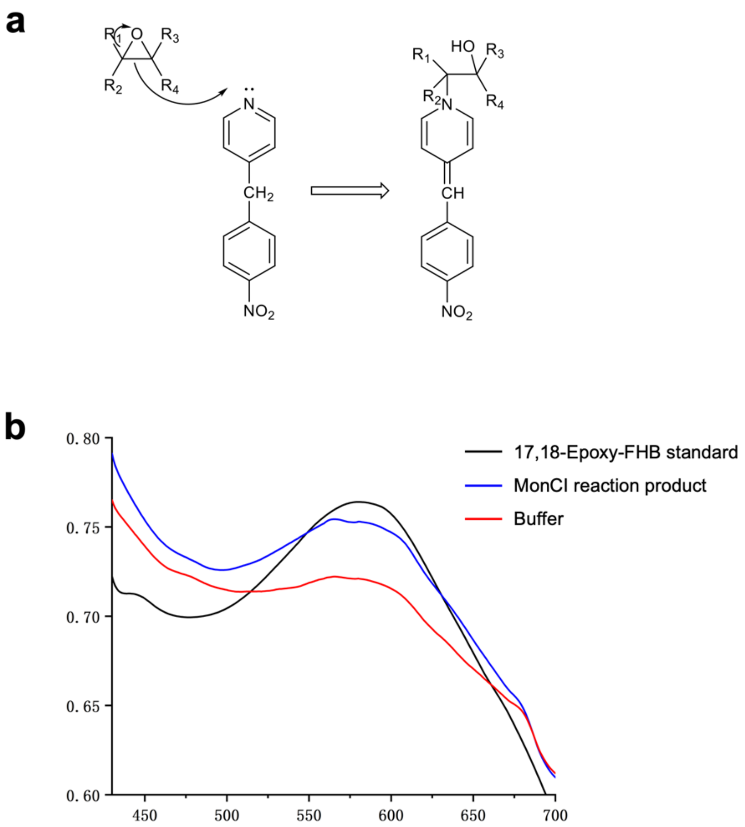

Supplementary Fig. 7 | Colorimetric assay for detection of epoxides. a Epoxide reacts with 4-(4-nitrobenzyl)pyridine (NBP) to form a purple-colored colored solution, which can be monitored spectrophotometrically. **b** NBP reacted 17,18-Epoxy-FHB standard and NBP reacted MonCl reaction product both display UV absorbance peak at 580 nm. Source data are provided as a Source Data file.

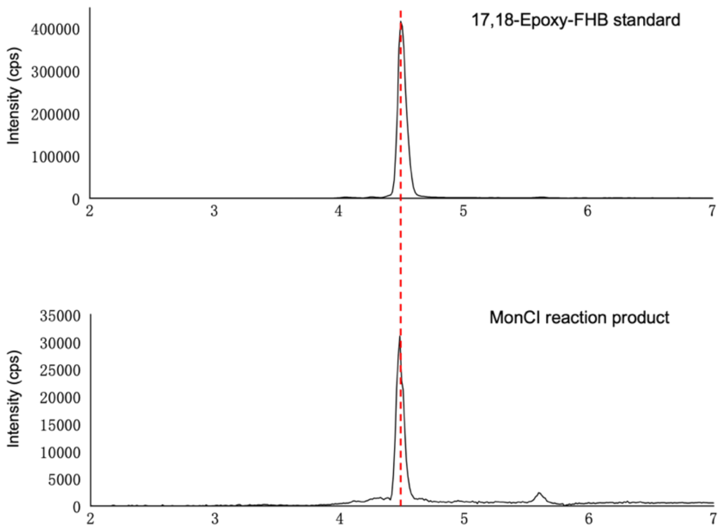

Supplementary Fig. 8 | LC-MS analysis of the 17,18-epoxy-FHB standard and MonCl reaction product. The synthesized 17,18-epoxy-FHB standard (top) has a retention time of 4.49 minutes, and the enzyme reaction product (bottom) has a retention time of 4.48 minutes. Source data are provided as a Source Data file.

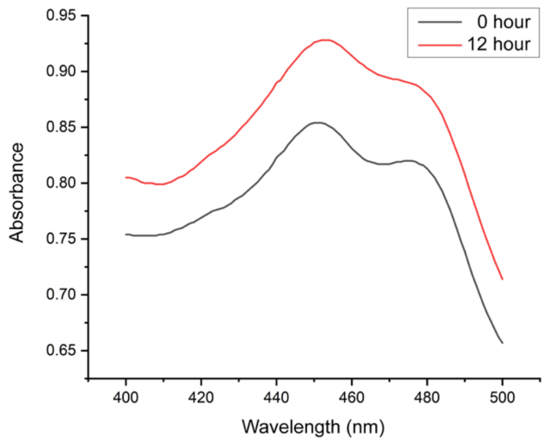

Supplementary Fig. 9 | UV-Vis spectrum of MonCI before and after catalysis. MonCI solution does not have an absorption peak at 460 nm which is characteristic of flavin-N5-oxide, a reaction intermediate that has been detected in some monooxygenases. Source data are provided as a Source Data file.

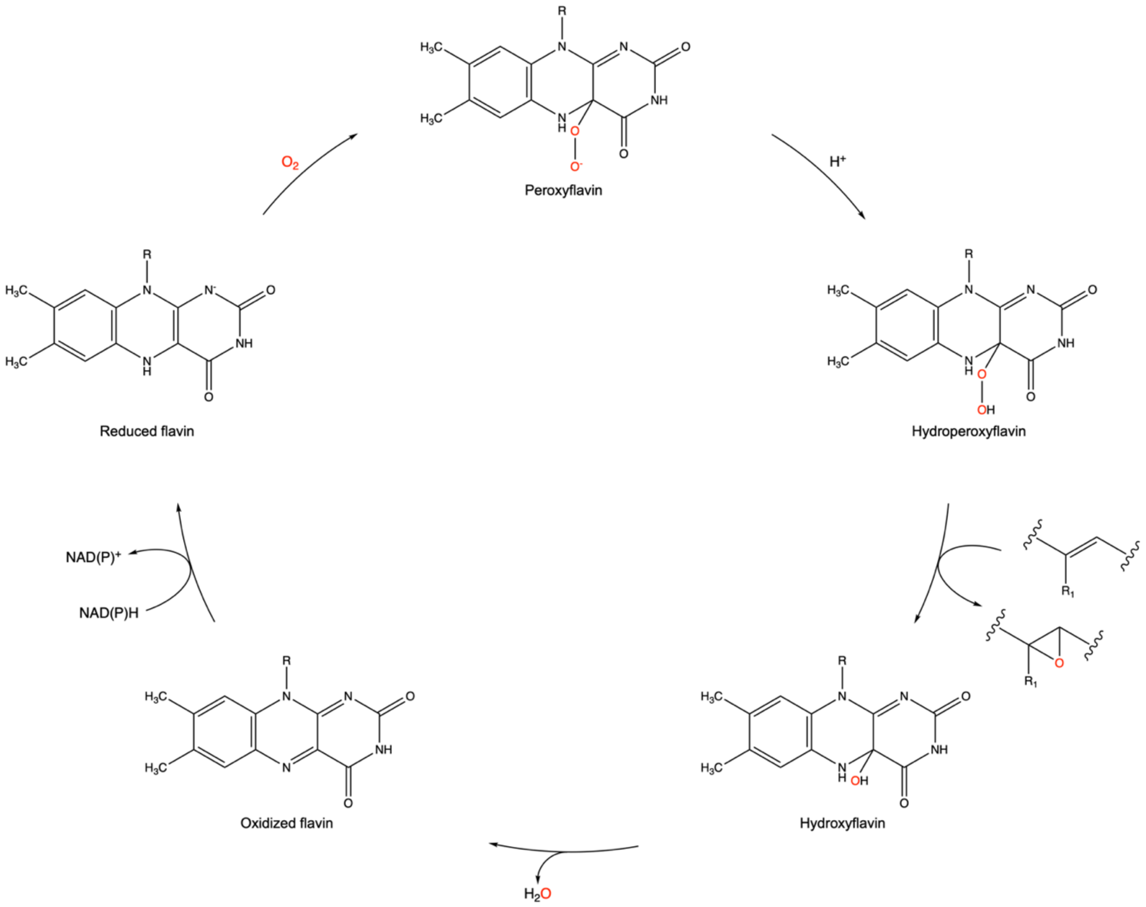

Supplementary Fig. 10 | Proposed mechanism of MonCI catalyzed epoxidation reaction. Oxidized FAD is reduced by NAD(P)H, which subsequently reacts with oxygen and proton to generate the hydroperoxylflavin intermediate. During the epoxidation reaction, the distal oxygen atom of the intermedaiate is transferred to the substrate and hydroxylflavin is formed. Finally, removal of water regenerates the oxidized flavin.

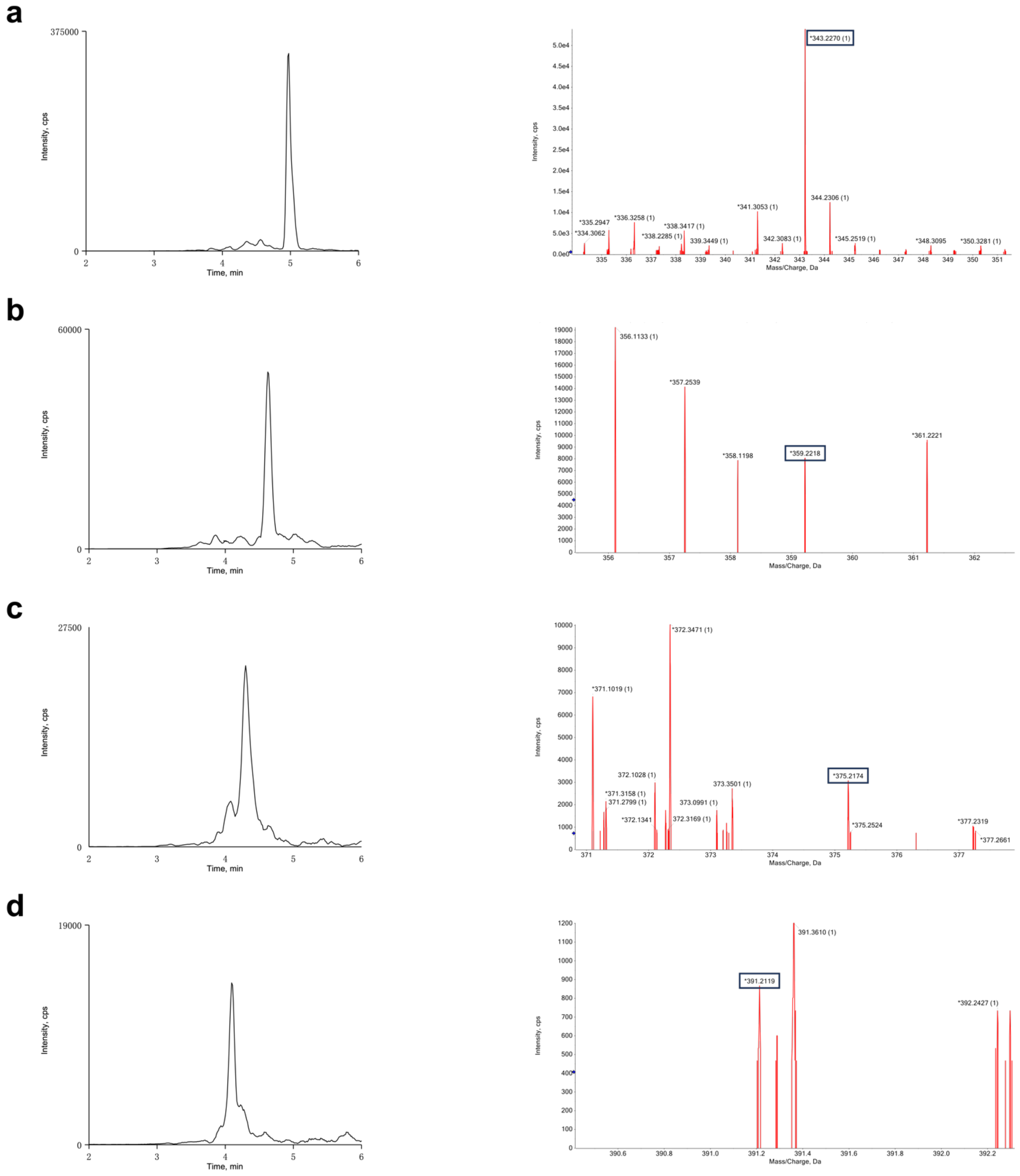

Supplementary Fig. 11 | LC-MS characterization of farnesyl 4-hydroxybenzoate and isolated epoxidation products formed by MonCI under the NADH regeneration condition. **a** Farnesyl 4-hydroxybenzoate (theoretical $[M\text{-}H]^+$ = 343.2267 m/z). **b** Monoepoxide product (theoretical $[M\text{-}H]^+$ = 359.2216 m/z). **c** Bisepoxide product (theoretical $[M\text{-}H]^+$ = 375.2166 m/z). **d** Triepoxide product (theoretical $[M\text{-}H]^+$ = 391.2116). Source data are provided as a Source Data file.

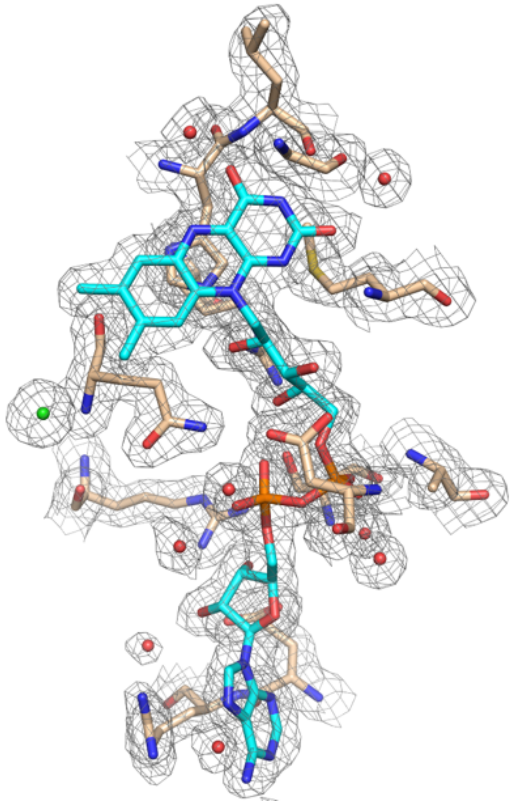

Supplementary Fig. 12 | 2*F*o-*F*c map of FAD and surrounding residues contoured at 1.1σ. Chloride ion and water molecules are represented as green and red spheres, respectively.

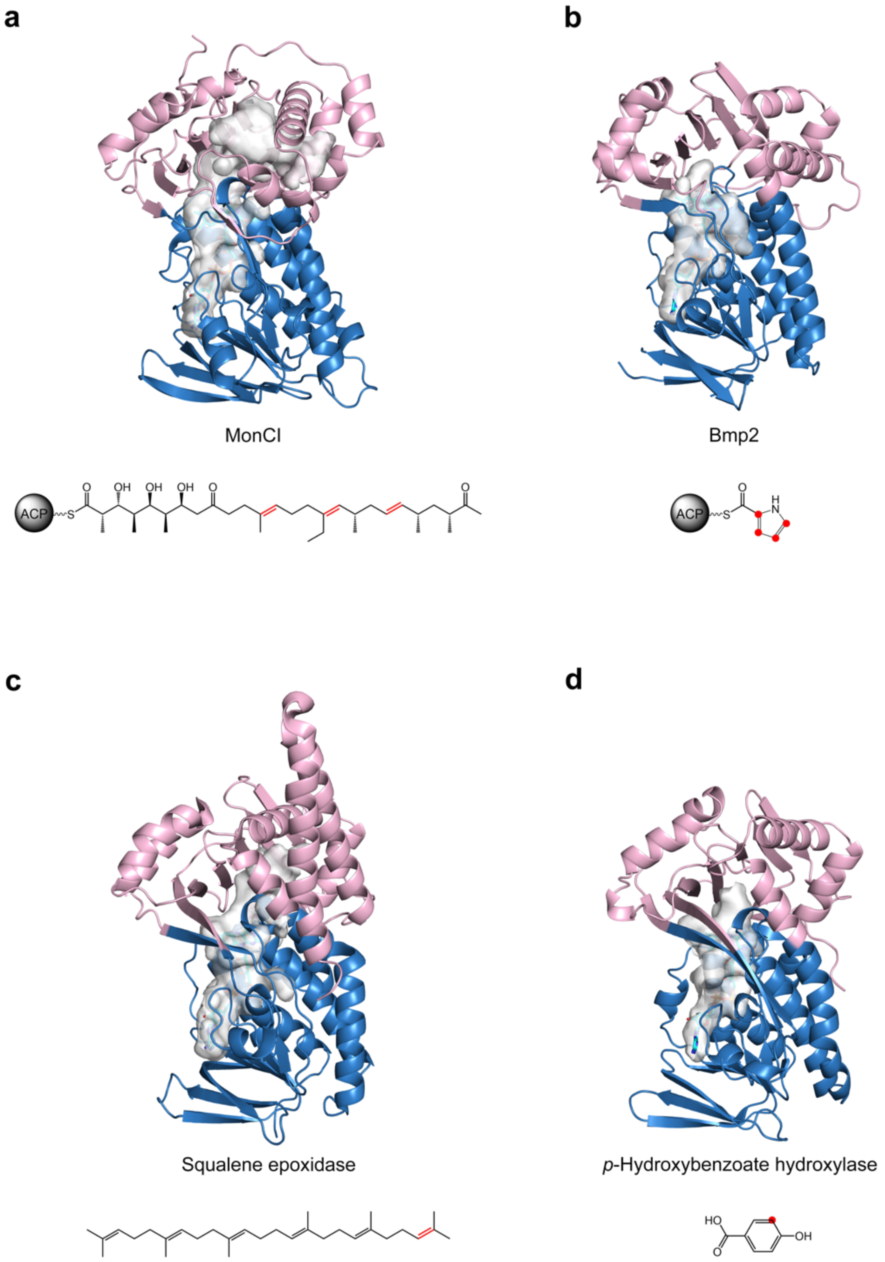

Supplementary Fig. 13 | X-ray crystal structure of MonCI and its structural homologs and the chemical structure of the respective substrates. The FAD-binding domain of each protein is colored in blue and the substrate-binding domain is colored in pink. The fused FAD- and substrate-binding pocket is shown in grey. Site(s) of chemical transformation in each substrate molecule is highlighted in red. ACP: Acyl carrier protein. **a** MonCI from *Streptomyces cinnamonensis* (PDB ID: 8T3P). **b** Bmp2 from *Marinomonas mediterranea* (PDB ID: 5BVA). **c** Human squalene epoxidase (PDB ID: 6C6N). **d** *p*-Hydroxybenzoate hydroxylase from *Pseudomonas fluorescens* (PDB ID: 1PBE).

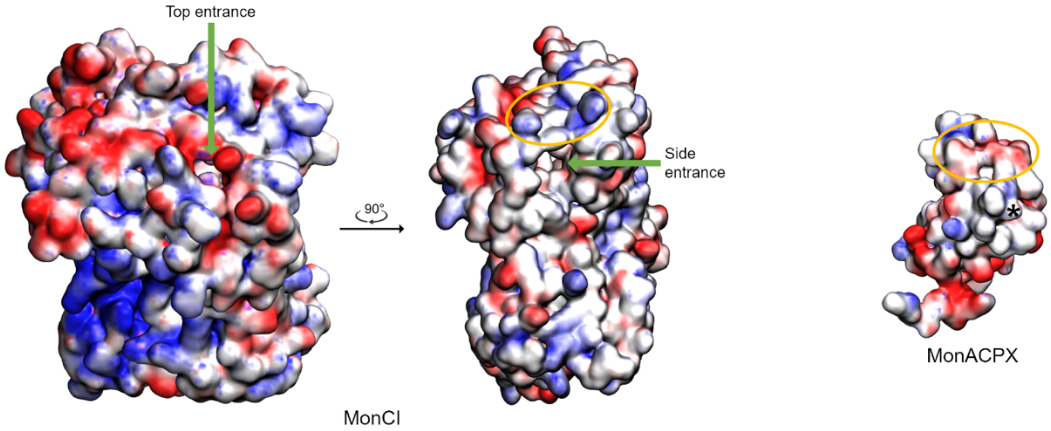

Supplementary Fig. 14 | Electrostatic surface potential map of MonCI and MonACPX calculated using the VMD and APBS program. Color scale is from -4 kbTec-1 (red) to 4 kbTec-1 (blue). Colors range from blue (positive, +0.12) to white (neutral, 0) to red (negative, -0.12). The yellow circles indicate the shared contact surface area between MonCI and MonACPX predicted by molecular docking. Asterix denotes the location of Ser60 in MonACPX.

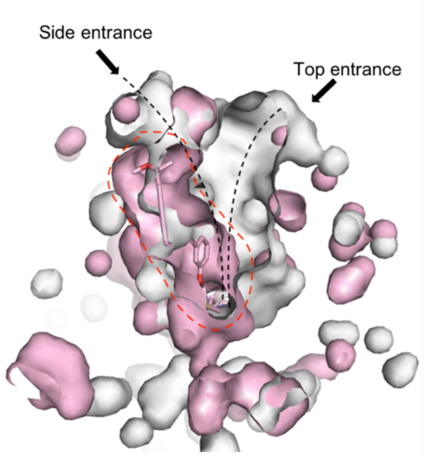

Supplementary Fig. 15 | Substrate-binding pocket alignment of MonCI and human squalene epoxidase (PDB ID: 6C6N). The pocket surface of MonCI is shown in white and the pocket surface of SQLE is shown in pink. The squalene epoxidase-bound Cmpd-4 inhibitor is shown as a stick model and the binding pocket is marked with the red broken line. The two entrances in MonCI and the respective trajectory leading to the active site are marked with black broken lines.

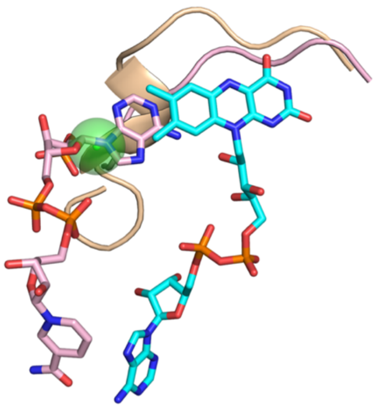

Supplementary Fig. 16 | Alignment of the MonCl and the 4-hydroxybenzoate hydroxylase (PDB ID: 1K0J) crystal structure. For MonCl, the chloride ion (green sphere), FAD (cyan stick model), and the NAD(P)H interacting segment (gold ribbon) are shown. For 4-hydroxybenzoate hydroxylase, the NAD(P)H binding loop (pink ribbon) the NADPH (pink stick model) cofactor is shown.

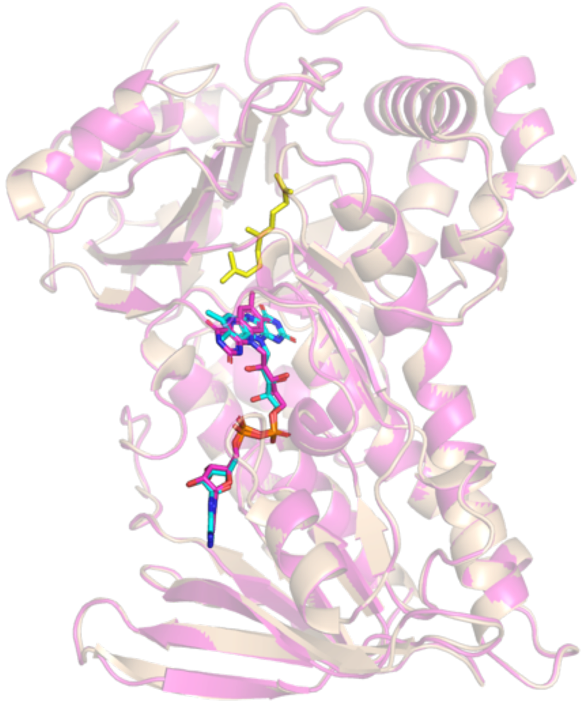

Supplementary Fig. 17 | Predicted mode of FAD binding in MonCl. Overlay of the MonCl-FAD_{in} crystal structure (MonCl = gold, FAD_{in} = cyan) and the predicted MonCl-FAD_{out} structure (MonCl = pink, FAD_{out} = pink).

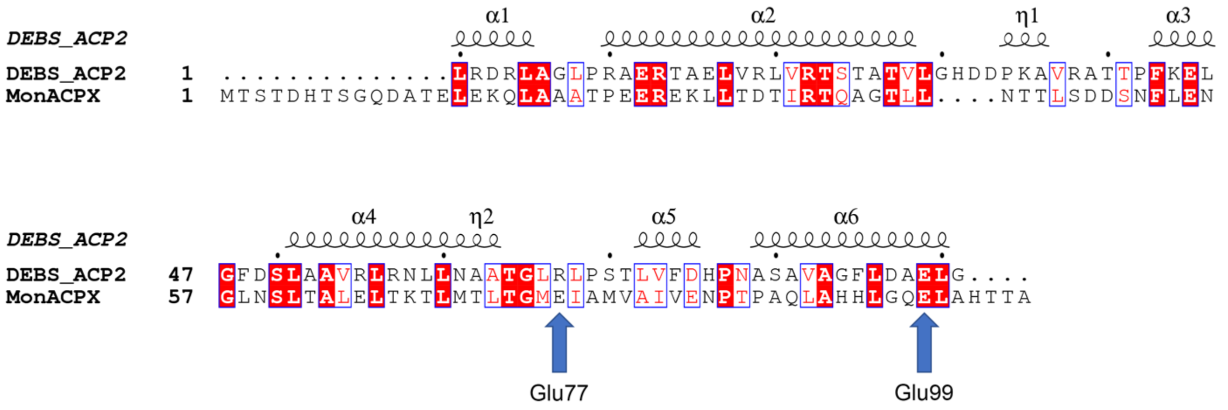

Supplementary Fig. 18 | Amino acid sequence alignment of MonACPX and deoxyerythronolide B synthase ACP2. Glu77 and Glu99 of MonACPX participate in salt bridge formation with Arg412 and Arg454 of MonCI, respectively.

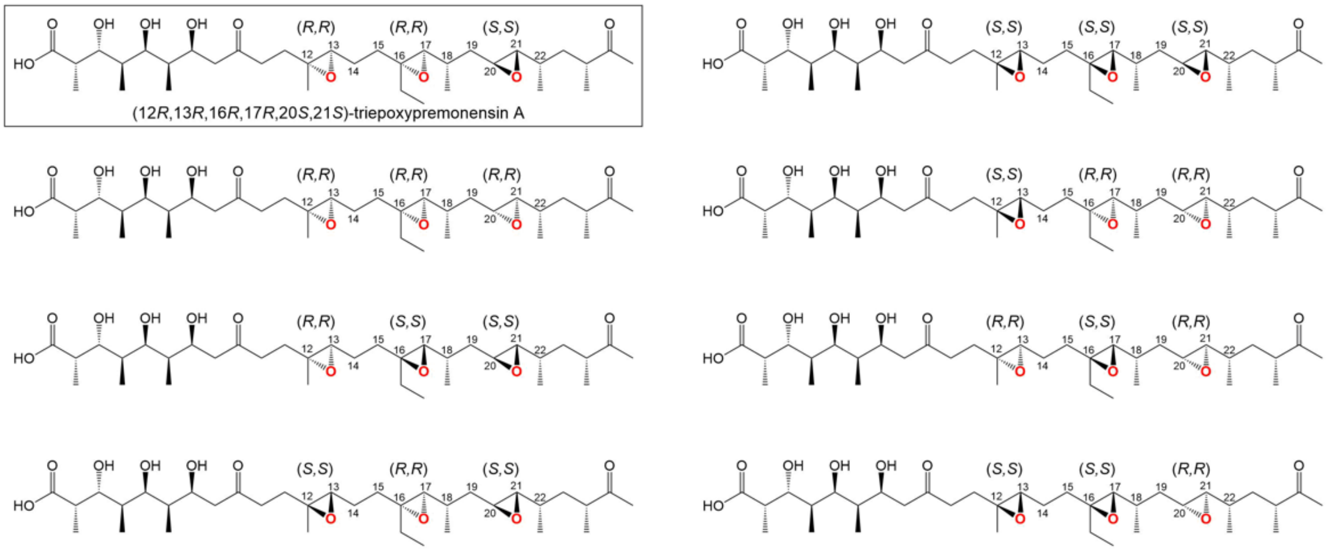

Supplementary Fig. 19 | Triepoxypremonensin A stereoisomers. Each of the three C=C groups in premonensin A can be converted to an (*R*,*R*)- or (*S*,*S*)-epoxide. Therefore, a maximum of eight different triepoxypremonensin A products can be potentially generated.

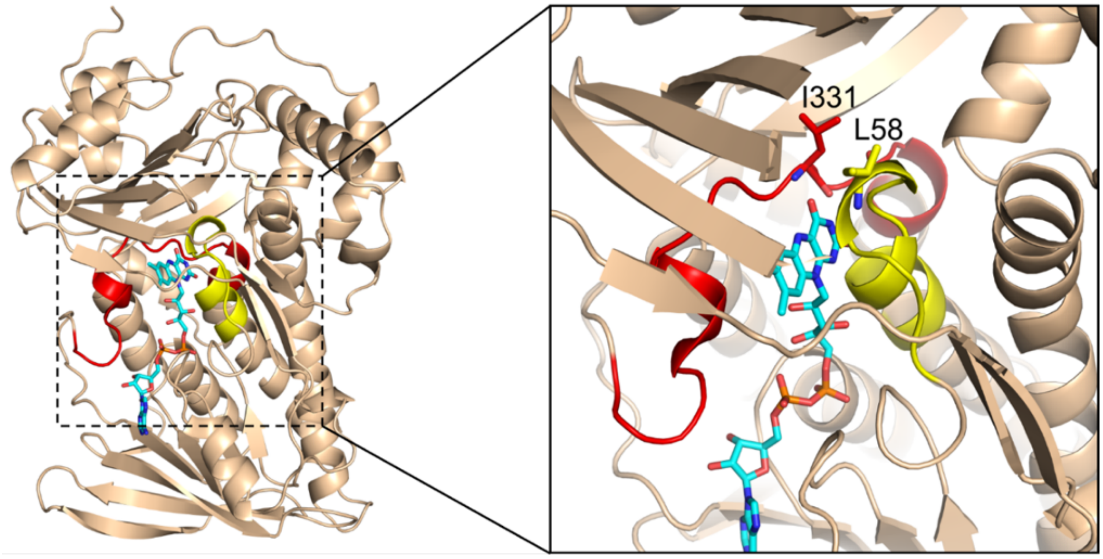

Supplementary Fig. 20 | X-ray crystal structure of MonCI. The conserved sequence RKGXPQXRHXHXLW (residues 47-64) is highlighted in red, and the conserved sequence AFNPXXGHGMSXXA (residues 327-340) is highlighted in yellow. FAD, Leu58, and Ile331 are shown in stick representation.

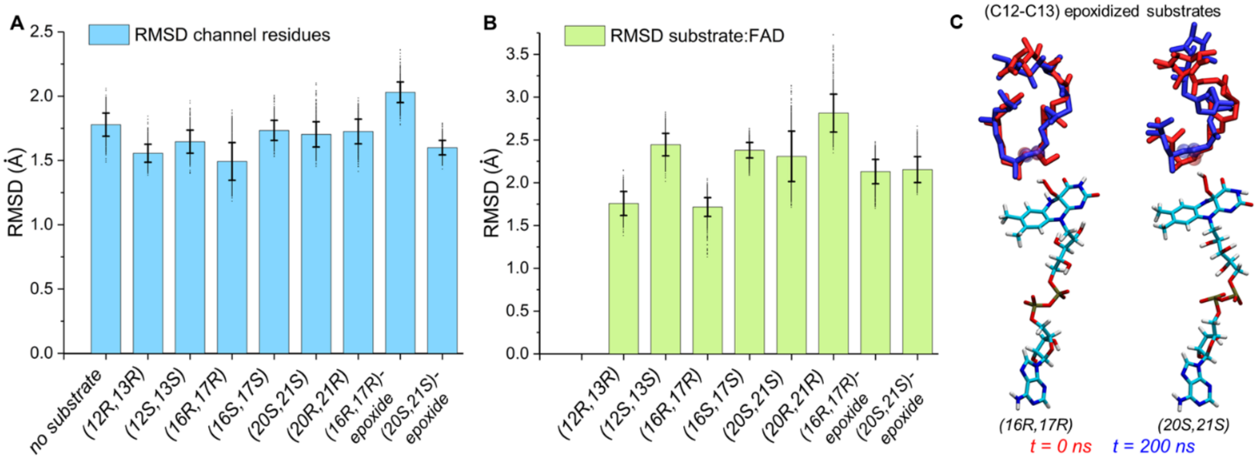

Supplementary Fig. 21 | Molecular dynamics calculations on substrate-FAD-4a-OOH complexes. a RMSD values of channel residues (defined in methods) for all systems. The systems labeled as (12*R*,13*R*), (12*S*,13*S*), (16*R*,17*R*), (16*S*,17*S*), (20*S*,21*S*), (20*R*,21*R*) initially had the substrates in their starting conformations. The systems labeled as (16*R*,17*R*)-epoxide and (20*S*,21*S*)-epoxide refer to (12*R*,13*R*)-monoepoxide substrate molecules in conformations where either the (16*R*,17*R*) or (20*S*,21*S*) C=C bonds are initially near the distal oxygen atom of C(4a)-hydroperoxy group. Data are presented as mean values ± SD (n = 1,000 structures extracted from the last 100 ns of one MD trajectory). **b** RMSD values of substrate-FAD-4a-OOH complexes for all systems. Data are presented as mean values ± SD (n = 1,000 structures extracted from the last 100 ns of one MD trajectory). **c** Conformation of C12-C13 epoxidized substrates at the start (red) and after 200 ns (blue) of simulations. The color scheme is the same as in Fig. 8.

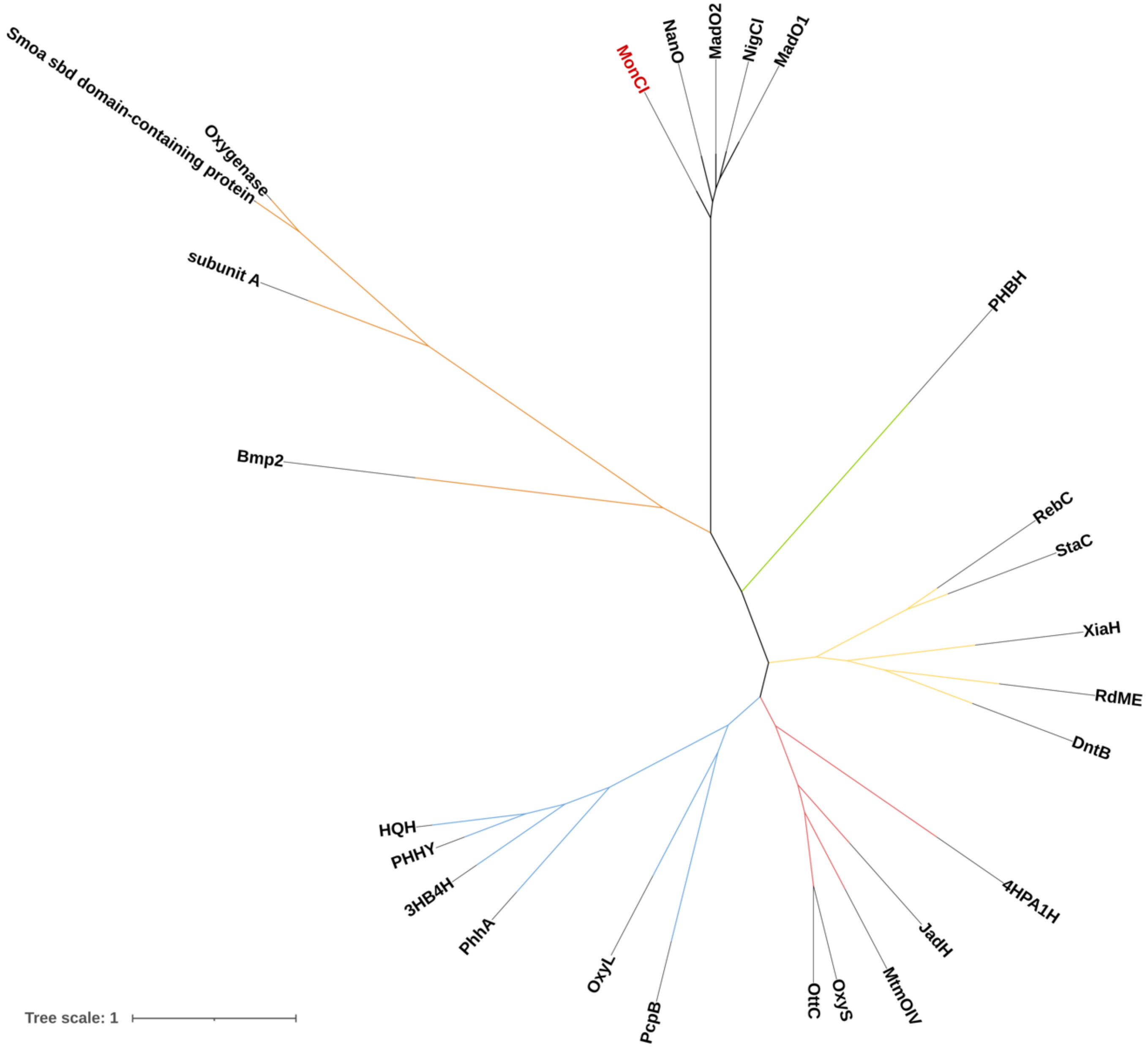

Supplementary Fig. 22 | Condensed phylogenetic analysis of flavin-dependent monooxygenases. Visualization of the phylogenetic relationships using a bootstrapped distance tree (1,000 iterations).

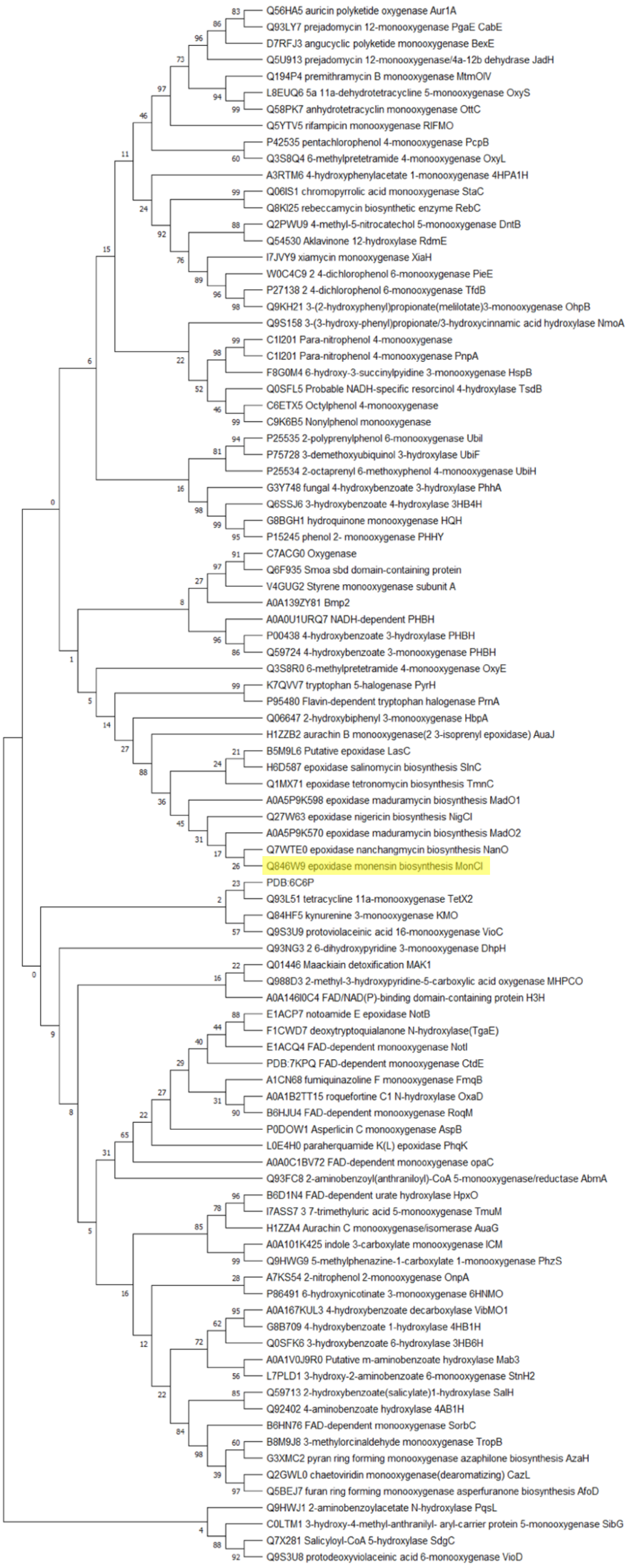

Supplementary Fig. 23 | Comprehensive phylogenetic analysis of flavin-dependent monooxygenases. The Bootstrap co-evolutionary tree was constructed by amino acid sequence alignment using the ClustalW method in MEGA11, and then generated using the neighbor-joining method. The evolutionary distance was calculated using the JTT matrix, and the proteins were analyzed by iterating 1,000 times.
